# Assessing the impact of the 4CL enzyme complex on the robustness of monolignol biosynthesis using metabolic pathway analysis

**DOI:** 10.1371/journal.pone.0193896

**Published:** 2018-03-06

**Authors:** Punith Naik, Jack P. Wang, Ronald Sederoff, Vincent Chiang, Cranos Williams, Joel J. Ducoste

**Affiliations:** 1 Civil, Construction, and Environmental Engineering, North Carolina State University, Raleigh, North Carolina, United States of America; 2 Forest Biotechnology Group, Department of Forestry and Environmental Resources, North Carolina State University, Raleigh, North Carolina, United States of America; 3 Electrical and Computer Engineering, North Carolina State University, Raleigh, North Carolina, United States of America; USDA Forest Service, UNITED STATES

## Abstract

Lignin is a polymer present in the secondary cell walls of all vascular plants. It is a known barrier to pulping and the extraction of high-energy sugars from cellulosic biomass. The challenge faced with predicting outcomes of transgenic plants with reduced lignin is due in part to the presence of unique protein-protein interactions that influence the regulation and metabolic flux in the pathway. Yet, it is unclear why certain plants have evolved to create these protein complexes. In this study, we use mathematical models to investigate the role that the protein complex, formed specifically between Ptr4CL3 and Ptr4CL5 enzymes, have on the monolignol biosynthesis pathway. The role of this Ptr4CL3-Ptr4CL5 enzyme complex on the steady state flux distribution was quantified by performing Monte Carlo simulations. The effect of this complex on the robustness and the homeostatic properties of the pathway were identified by performing sensitivity and stability analyses, respectively. Results from these robustness and stability analyses suggest that the monolignol biosynthetic pathway is resilient to mild perturbations in the presence of the Ptr4CL3-Ptr4CL5 complex. Specifically, the presence of Ptr4CL3-Ptr4CL5 complex increased the stability of the pathway by 22%. The robustness in the pathway is maintained due to the presence of multiple enzyme isoforms as well as the presence of alternative pathways resulting from the presence of the Ptr4CL3-Ptr4CL5 complex.

## Introduction

Lignin is the second most abundant complex polymer that is synthesized in the secondary cell walls of all vascular plants [1]. It enables the transport of water and nutrients through the stem, provides upright growth, and the protection against pathogens. Recalcitrance of lignin hinders the usage of plant biomass as a viable source of biofuels or in the use of plant biomass in pulp and paper. The lignin polymer is primarily composed of derivatives of the H, G, and S monolignols, which are synthesized via the phenylpropanoid pathway [2]. The structure of the lignin polymer is directly linked to variations in lignin content and composition. Therefore, the removal of lignin for the above uses requires either costly chemical pre-treatment or strong knowledge of the regulatory and metabolic framework to influence its formation [3,4].

The lignin biosynthetic pathway consists of twenty-four metabolites, twenty-one enzymes belonging to ten gene families, and thirty-five reactions that convert various reactants to products (S1 Fig). The reactions and the enzymes catalyzing each of the reactions are outlined in S1 Table. Substantial effort has been directed towards understanding the lignin biosynthesis pathway in plants to lower the amount of total lignin and/or change its relative ratio of monolignol subunits. Although all the metabolites, enzymes, and reactions involved in the monolignol biosynthesis has been identified, critical details about the pathway regulation, however, remain un-clear [5].

Previous attempts towards quantitatively analyzing the monolignol biosynthesis pathway primarily relied on the usage of a static based kinetic model (Flux Balance Analysis) [5,6]. The results from these analyses are largely hypothetical as the output is primarily based on constraints that are challenging to prove experimentally [7]. Results from prior simulations of the kinetic model for the metabolic network of monolignol biosynthesis [8] provided useful information about the role of various enzymes on the flux distribution within the pathway, as well as the role of individual enzymes on the lignin content and structure. However, the model did not incorporate the enzyme complex formation resulting from the interaction between Ptr4CL family enzymes. In another recently completed study on the lignin biosynthesis pathway, researchers revealed that Ptr4CL3 and Ptr4CL5 enzymes react with each other forming an enzyme-enzyme complex [9]. To date, an extensive analysis of the role of the Ptr4CL3-Ptr4CL5 complex in Populus trichocarpa’s lignin biosynthesis pathway has not been performed.

### Complex formation

It has been argued that for proper function, proteins can form complexes that may be composed of monomers or heteromers [10] and provide the plant with an evolutionary advantage. For the case of the monolignol biosynthetic pathway, it was experimentally determined that the 4CL enzymes that convert the various hydroxycinnamic acids into CoA esters, exhibited protein—protein interactions. Chemical crosslinking coupled with immune-detection and mass spectrometry suggested that the Ptr4CL3 and Ptr4CL5 are present at a ratio of 3:1 in a complex [9]. Using this information and assuming mass action kinetics, a mechanistic model was developed to quantify the rate of reaction resulting from the complex [9]. In single substrate reactions, Ptr4CL5 showed broader substrate affinity as compared to Ptr4CL3. Ptr4CL3 displayed competitive inhibition while Ptr4CL5 showed both allosteric regulation and substrate self-inhibition [11]. The rate equations describing the role of the complex were developed only for p-coumaric acid and caffeic acid, because Ptr4CL3 and Ptr4CL5 showed competitive and uncompetitive inhibition by substrates p-coumaric acid and caffeic acid. The rate equations were not developed for ferulic acid, 5-hydroxyferulic acid and sinapic acid since they were found to be weak inhibitors [9,11].

The results from the 4CL transgenic experiments suggest that the down regulation of 4CL leads to a reduction in lignin content in tobacco, Arabidopsis, and aspen [12,13,14] and to higher amounts of cell wall–bound hydroxycinnamic acids (p-coumaric, ferulic, and sinapic acids) in tobacco and poplar [12,13]. The effects of 4CL transgenics on S/G lignin composition are contradictory. In tobacco, a reduction in S units was reported [12,15] while in Arabidopsis, only G units were reduced [14]. In transgenic aspen, the S/G ratio was shown to be similar to that of the control (2:1) [13]. Previously, it was unclear why two alternative pathways exist for the 3-hydroxylation step in monolignol biosynthesis in *P*. *trichocarpa*. These results suggest that the exact role of 4CL on the lignin structure and composition is still fuzzy and a more detailed quantification of the effects of 4CL on the lignin biosynthetic pathway would greatly enhance our understanding of the role of the Ptr4CL3-Ptr4CL5 complex in the pathway.

In this work, we augmented and analyzed the mathematical model that was previously developed to simulate the lignin biosynthetic pathway [8] by incorporating the model developed to quantify the role of complex on the reaction rate [9]. Using this revised model, we posed several questions to assess the role of the complex: (1) How does the steady state flux and steady state metabolite concentration change over the range of nominal *in vivo* metabolite concentrations? (2) To what extent does the protein complex maintain or change the steady state flux and metabolite concentrations under the perturbations of other enzymes in the pathway? Finally, (3) Does the lignin biosynthetic pathway exhibit increased robustness/ homeostatic behaviour in the presence of the protein complex? We will explore how the Ptr4CL3-Ptr4CL5 complex may provide a mechanism to regulate these alternative pathway fluxes for the 3-hydroxylation step and provide important insights to robustness/homeostasis in lignin biosynthesis.

## Methodology

### Computing steady state metabolite concentration

Biological networks are characterized by highly nonlinear interactions between various components within the network. One of the results of these nonlinear interactions in biological networks is the presence of alternative steady states, which correspond to metabolic flux conditions that the cell achieves during growth or when perturbed by external stressors. For the monolignol biosynthetic pathway, the existence of alternative steady states corresponds to different phenotypes (lignin structure). The identification of alternative steady states would aid plant biologists in determining the biological conditions that would lead to a particular lignin structure. In particular, we anticipate that the modelling results will allow researchers to identify the regions (combination of Ptr4CL3/Ptr4CL5) where there is a high likelihood of observing changes in the lignin content or structure.

Identification of alternative steady states was performed using a Latin Hypercube Sampling (LHS) procedure. In LHS, random initial metabolite concentrations are sampled and used to simulate the system of Ordinary Differential Equations (ODE) to achieve a steady state concentration.

The above procedure was repeated with 10,000 different initial metabolite concentrations, thus enabling us to identify all possible steady states of the system. The ranges of initial sampled concentrations were specified based on the maximum K_m_ values for each substrate enzyme reaction (i.e., values of metabolites ranged from 0 to 10 times the K_m_ values) [8]. A uniform distribution and 10,000 different concentrations of the metabolites were sampled to perform the simulations.

The reaction fluxes for all the reactions in the pathway were expressed in the form of Michaelis Menten kinetics [8]. The rate equations developed by [9] that capture the interactions between Ptr4CL3, Ptr4CL5, and the Ptr4CL3/Ptr4CL5 complex were incorporated in the lignin biosynthesis pathway model from [8]. A final list of all flux equations are provided in S2 and S3 Tables.

### Sensitivity analysis of Ptr4CLs on the monolignol biosynthetic pathway

Sensitivity analysis provides a measure of the influence of model parameters on the model output [16]. We used global sensitivity analysis [17] by varying the concentrations of Ptr4CL3 and Ptr4CL5 from 0-WT levels to quantify the effect of perturbations on the steady state metabolite flux. The inputs were only the Ptr4CL3 and Ptr4CL5 enzyme concentrations. The concentrations of all the other enzymes were fixed at their WT concentrations. In this study, the variance decomposition method [18] was used. The algorithm divides the output variance into explained and unexplained variance. The explained variance is a result of the variations in the output because of variations in the input parameters.

In this study, we used Ptr4CL3 and Ptr4CL5 as the factors and the steady state flux (V_1_ to V_32_) as the output. Assuming uniform distribution, 10,000 values of Ptr4CL3 and Ptr4CL5 were randomly sampled using the LHS procedure. The range of the enzyme concentration was assumed to be ±50% of the WT concentrations. All remaining enzymes were fixed at their respective WT concentrations. The first order and total sensitivity index were calculated using *eFAST* technique, which is based on Fourier transforms [18].

### Stability analysis of the monolignol biosynthetic pathway

One of the important aspects of the biochemical pathway modelling is stability of the steady states resulting from the interconnected kinetic models. It has been hypothesized that metabolic stability is the key mechanism through which the biological systems maintain homeostasis. Since the overall goal of this study is to identify the role of the Ptr4CL3-Ptr4CL5 enzyme complex on the monolignol biosynthetic pathway, performing stability analysis on the kinetic model would provide insights about the role of complex on the change in the stability of the system under perturbation.

The local stability of steady states can be assessed using the linearization principle [19]. In the presence of multiple steady states, the Jacobian matrix is evaluated over a range of steady state metabolite concentrations and for each steady state concentration, the Eigenvalues are calculated. In order to assess the role of the complex, the concentrations of Ptr4CL3 and Ptr4CL45 were varied from 0 to WT levels. The multiple steady state were identified by solving the system of ordinary differential equations to zero and solving them using Newton’s non-linear solver in MATLAB^®^ [20]. The steady state metabolite concentrations for all 24 metabolites were calculated for each pair of Ptr4CL3 and Ptr4CL5 values and the Jacobian matrix was evaluated over the range of steady state concentrations. The Jacobian matrix was calculated numerically using the forward difference method and the Eigenvalues were calculated using the MATLAB *eig* function. More information about steady state analysis and its application on biochemical pathways can be found in [21,22,23].

## Results and discussion

### Steady state metabolite concentration variation under WT conditions

**Fig 1:**
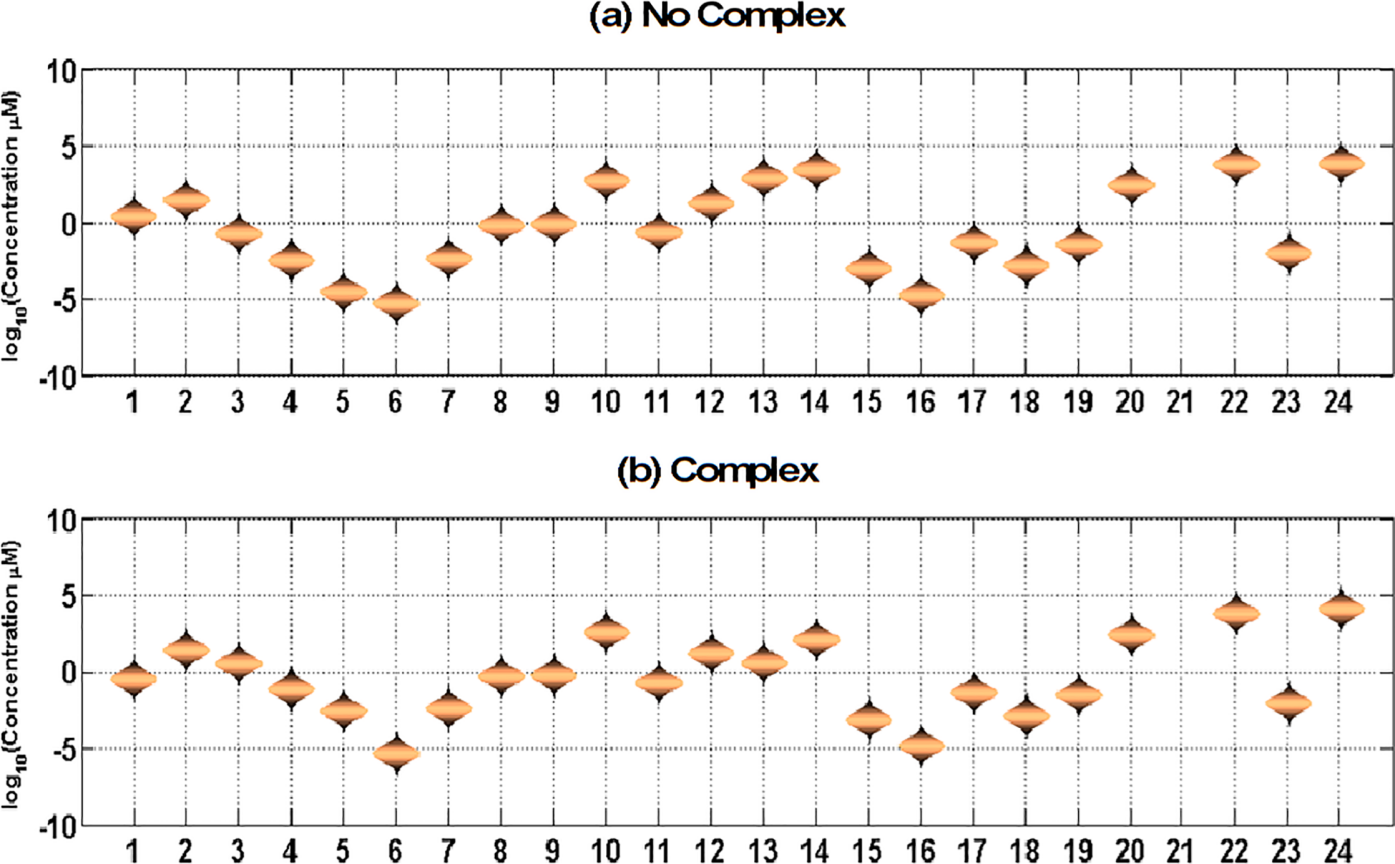
Steady state metabolite concentrations observed for the model (a) without the complex and (b) model with the complex under WT enzyme concentrations. The concentrations are reported in log scale because of the variability in the stable steady state concentrations for different metabolites. The distribution of steady state values is a result of 10,000 runs performed under varying initial concentrations of the metabolites.

The distribution of steady state concentrations of the 24 metabolites with and without complex resulting from 10000 random samples of initial metabolite concentrations are shown in Fig 1. In Fig 1, the model predicts that all the metabolite concentration distributions in the pathway are similar (i.e., within 1 fold change difference) in the absence and presence of Ptr4CL3-Ptr4CL5 complex except for *p*-coumaric acid, caffeic acid and ferulic acid. The steady state concentration of p-coumaric acid, caffeic acid, and ferulic acid in the presence of the complex are 20 fold, 24 fold, and 100 fold higher, respectively, than for the model without the complex. The primary reason for the increased steady state concentration in the presence of complex is because the substrate specificity of Ptr4CL3 is higher for the acids as compared to Ptr4CL5, as well as the self-inhibition of caffeic acid in the presence of Ptr4CL5. In the presence of the complex, the inhibitory effect of ferulic acid on p*-*coumaric acid and caffeic acid is higher, due to the accumulation of ferulic acid, hence resulting in an accumulation of p*-*coumaric acid and caffeic acid [9]. As with the steady state concentrations, the steady state flux distributions are identical to the model without the complex except for the steady state fluxes V_3_ (p-coumaric acid to caffeic acid), V_7_ (p-coumaric acid to 4-coumaroyl-CoA) and V_8_ (caffeic acid to caffeoyl-CoA). The total lignin content and composition also remained unchanged. These results support the recent findings that have demonstrated that lignin biosynthesis is more resistant to perturbations [8], In their study, Wang et al. [8] reported that most enzymes are produced in excess of what is required for a normal lignin phenotype, and a significant reduction in enzyme quantity (>50% of wild type) is needed to affect lignin. This resistance to perturbation is also due to redundancies in the monolignol biosynthesis pathway where six of the ten enzyme families have functional redundancies [24].

**Fig 2:**
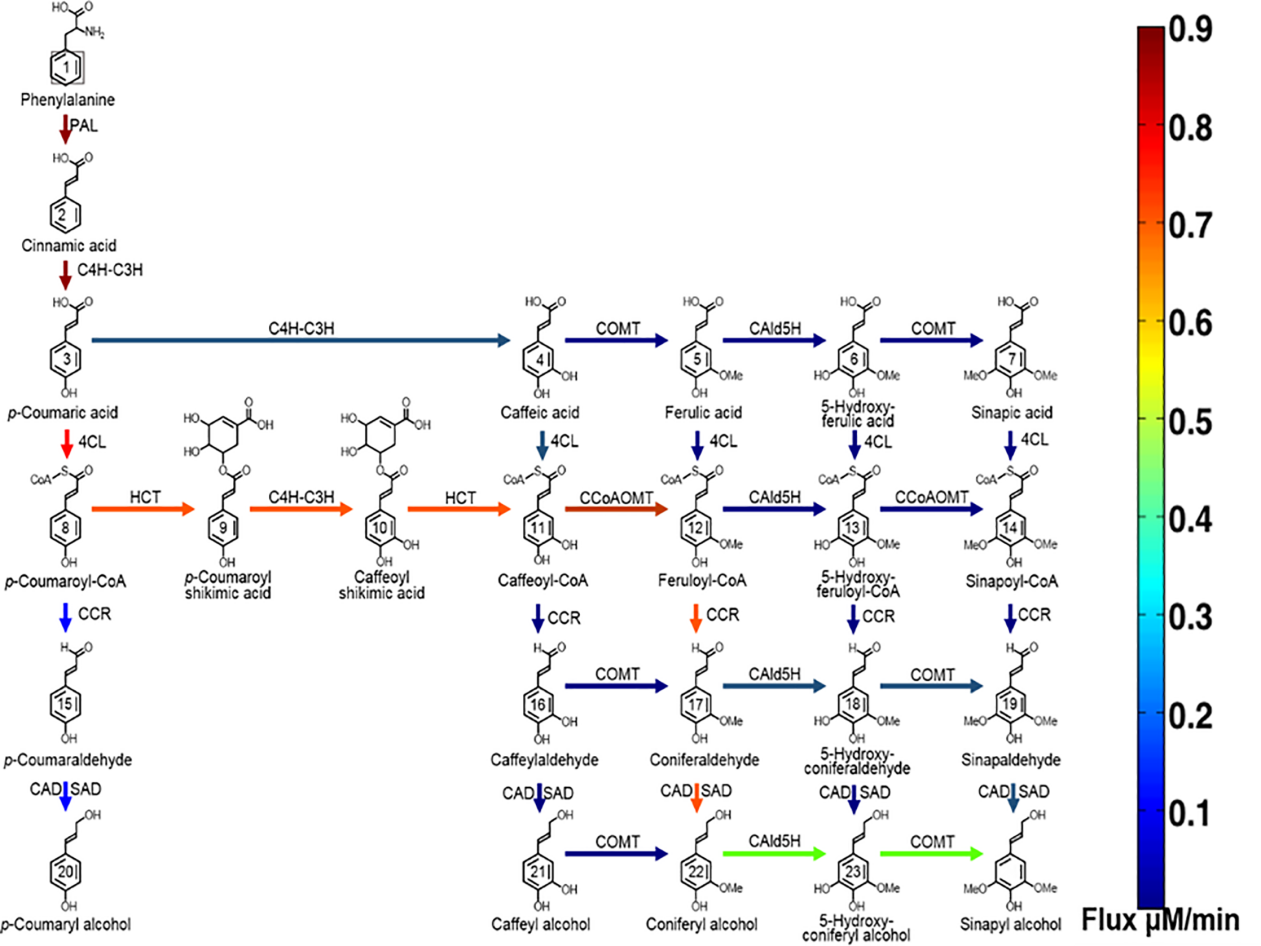
Steady state flux pattern observed for the model without the complex WT enzyme concentrations. Colored arrows represent the magnitude of flux and the colors can be mapped to their flux values with the color bar.

**Fig 3:**
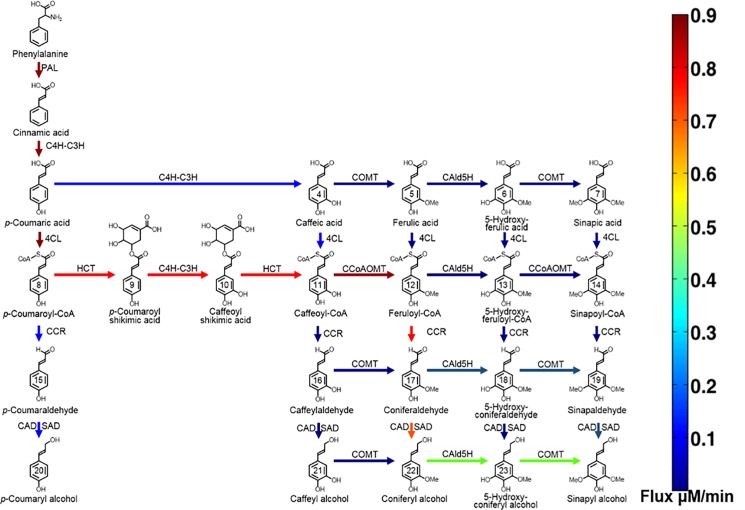
Steady state flux pattern observed for the model with the complex under WT enzyme concentrations colored arrows represent the magnitude of the flux as shown in the color bar.

The resulting steady state flux and the preferred pathway for monolignol biosynthesis in the presence and absence of the complex under WT conditions are shown in Figs 2 and 3, respectively. Under WT conditions, the presence of the Ptr4CL3-Ptr4CL5 complex provides an additional path for CoA ligation, where caffeic acid is also a preferred substrate of Ptr4CL in addition to p*-*coumaric acid. In the absence of the Ptr4CL3-Ptr4CL5 complex, the p*-*coumaric acid substrate specificity of Ptr4CL3 is 4-fold higher than Ptr4CL5. In the presence of the Ptr4CL3-Ptr4CL5 complex, the majority of the flux in the pathway is routed through p*-*coumaric acid with a small portion of the flux flowing through caffeic acid (300% increase). The small portion of the flux that flows from p-coumaric acid to caffeic acid and caffeic acid to caffeoyl-CoA as observed for the case of the Ptr4CL3-Ptr4CL5 complex is due to the controlling role of Ptr4CL5 in lignin formation. Ptr4CL5 in the complex controls the amount of activation and inhibition [9,11].

### Role of Ptr4CL3-Ptr4CL5 complex on the flux distribution when Ptr4CL enzymes are perturbed

In this section, we computationally assessed the role of Ptr4CL3-Ptr4CL5 complex on the lignin biosynthesis pathway in the presence of enzymatic perturbations. The enzyme concentration of Ptr4CL3 and Ptr4CL5 were varied from 0 to WT (Ptr4CL3(1.4):Ptr4CL5(0.12)) and 10,000 pairs of different combinations of Ptr4CL3 and Ptr4CL5 concentrations were randomly selected. For each pair, the resulting steady state concentrations of the twenty-four metabolites were calculated.

Because the Ptr4CLs primarily mediate the conversion of hydroxycinnamic acids to their CoA derivatives, which are represented by the reactions V_7_ to V_11_, we assessed the localized effect of varying Ptr4CL concentrations on the reaction flux when subjected to enzymatic perturbation. Prior research showed that the fluxes V_10_ and V_11_ did not contribute towards monolignol biosynthesis [11]. The variation of reaction flux V_7_ as a function of total Ptr4CL in the absence of the Ptr4CL3-Ptr4CL5 complex is shown in S2 Fig. In absence of the complex under WT concentrations, the flux through the pathway is primarily routed through V_7_ (i.e., conversion of 4 coumaric acid to 4-coumaroyl-CoA); hence the steady state flux, V_7_, corresponds to the input flux 0.9 μM/min and the flux V_8_ (i.e., conversion of caffeic acid to cafferoyl-CoA) and V_9_ (i.e., conversion of ferulic acid to feruloyl-CoA) are 0 μM/min. As the concentration of total Ptr4CL3 and Ptr4CL5 are reduced from WT enzyme concentrations, we observe a decrease in the steady state flux V_7_ with a corresponding increase in flux V_8_. At very low concentrations, we clearly see a reduction in flux V7, V8 and V9.

The presence of Ptr4CL3-Ptr4CL5 complex results in more variation in the steady state flux V7,V8 and V9 as seen in S3 Fig. This variation in flux levels was not observed in the simulations performed without the complex. The turndown behaviour described earlier for simulations without the complex was not influenced by a specific combination of Ptr4CL3 and Ptr4CL5 but only the total amount of Ptr4CL protein. However, for the kinetic model with the complex, we observe that there is a range of values for each flux pathway V7, V8, and V9. In that simulation, it does matter which of the Ptr4CL proteins is controlled and its amount. When the complex is presented, the plant is able to adjust the flux profile within each total Ptr4CL range. While much of the mass flux is still routed through V7, there can be a significant amount of flux through V8 and some through V9. It suggests that the development of the complex allows the plant to form product from V7, V8, or V9 pathways and therefore produce a sustained formation of a specific lignin content and structural type even if there was some external stress that might lead to the reduction of either Ptr4CL3 or Ptr4CL5. That stress would result in a shift from V7 to V8 if no complex was produced as observed in S2 Fig since only the total Ptr4CL protein would control the mass flux through a specific pathway.

Two main feedback inhibitions primarily affect the steady state flux distribution when the Ptr4CL3 and Ptr4CL5 concentrations are perturbed between 0 and WT: (1) Caffeic acid inhibits the rate of conversion of p-coumaric acid to p-coumaroyl-CoA, and (2) Ferulic acid inhibits the rate of conversion of p-coumaric acid to p-coumaroyl-CoA (S4 Fig). The steady state distribution of metabolic flux V_7,_ V_8_ and V_9_ is shown in S5 Fig. The violin plot displays the distribution of the steady state flux and assumes that the steady state flux follows a normal distribution. The presence of two distinct flux regimes is observed for flux V_7_. The two flux regimes were generated from the presence of the bimodal steady state distribution of the caffeic and ferulic acid concentrations (S6 Fig). The high concentration of Ptr4CL5 results in low steady state concentrations of caffeic acid and ferulic acid in S6 Fig. The reduction in the concentration of Ptr4CL5 results in an accumulation of caffeic acid and ferulic acid as seen in S6 Fig.

**Fig 4:**
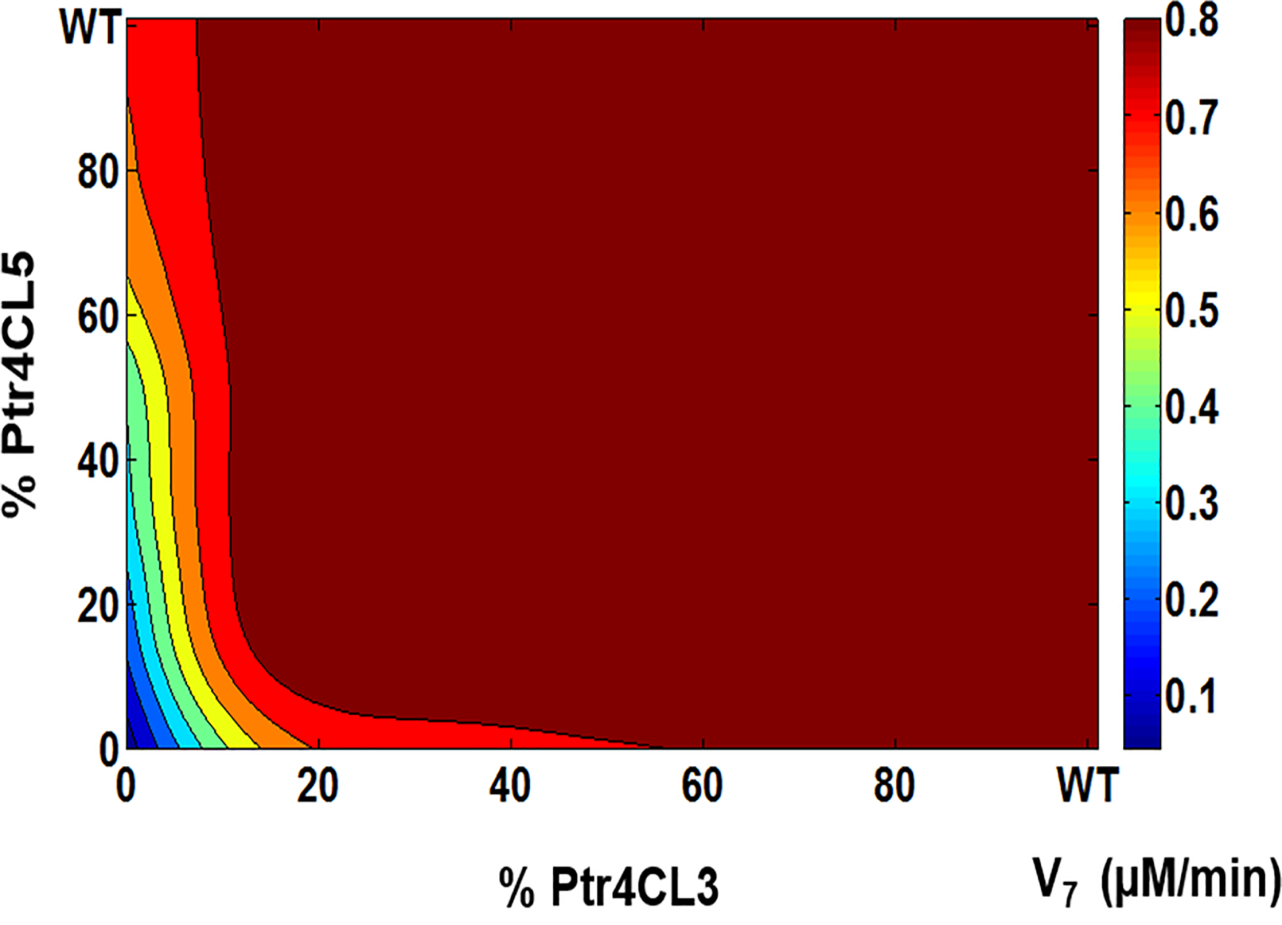
Contour plot showing the variation of steady state flux (V_7_) as a function of Ptr4CL3 and Ptr4CL5 concentration in the absence of a complex. The axis values represents the percentage of the protein concentration as a function of the wild type concentration. The color bar represents the flux values (μM/min).

**Fig 5:**
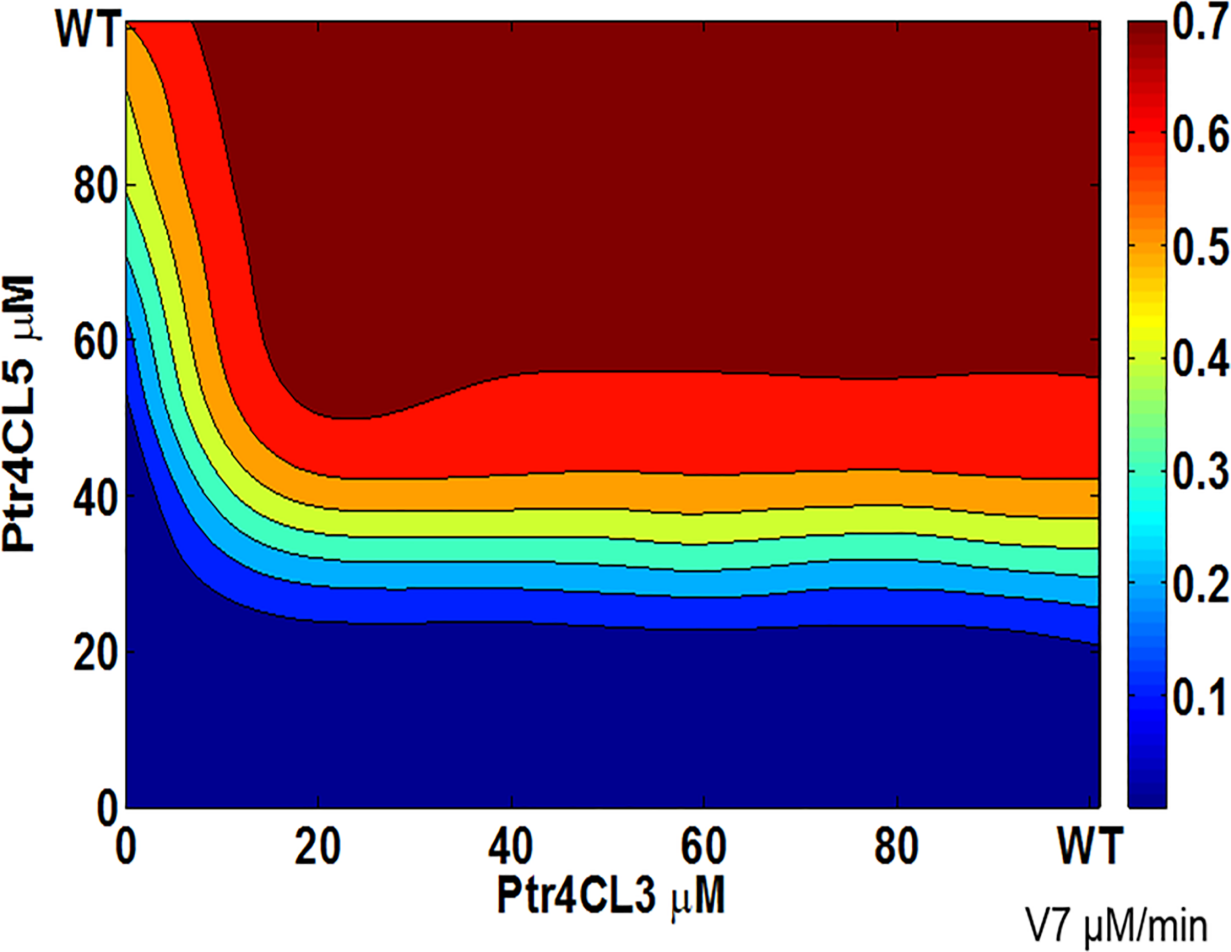
Contour plot showing the variation of steady state flux (V_7_) as a function of Ptr4CL3 and Ptr4CL5 concentration in the presence of a complex. The axis values represents the percentage of the protein concentration as a function of the wild type concentration. The color bar represents the flux values (μM/min).

The role of individual enzymes on the steady state flux distribution of V_7_ can be visualized with the help of contour plots presented in Fig 4 and Fig 5. In the absence of the Ptr4CL3-Ptr4CL5 complex, the changes in steady state flux (V_7_) is primarily brought about by changes in levels of Ptr4CL3 as seen in Fig 4. Under WT levels of Ptr4CL3 and Ptr4CL5, the steady state flux corresponds to a maximum value of 0.8 μM/min shown, which represents almost 75% of the steady state solutions. In order to observe a change in the steady state flux by 0.1 μM/min unit, a reduction in the Ptr4CL3 concentration of up to 90% of the WT levels would be required and achieved by holding the Ptr4CL5 concentration at WT levels. On the other hand, if the concentration of Ptr4CL5 were reduced by 90% of its WT concentration, then the concentration of Ptr4CL3 would have to be reduced by 80% of the WT concentration to observe a 10% reduction in the steady state flux. These results suggest that in the absence of Ptr4CL3 –Ptr4CL5 complex, the model is robust to perturbations, requiring a high degree of enzymatic adjustments to observe a significant change in the steady state flux. The robustness is primarily due to the abundance of Ptr4CL3 levels when compared to Ptr4CL5 levels. These results provide a mechanistic explanation for the severe downregulation of Ptr4CL genes to affect lignin biosynthesis in transgenic *P*. *trichocarpa* [8]

Prior experiments have suggested that the lignin structure and composition can be altered by targeting certain genes in the pathway. The model without complex suggests that the lignin composition and structure can only be altered under extreme perturbations, which is not consistent with the experimental studies reported in the literature. Robustness is a good trait since the system is able to endure changes without adapting. However, such robustness are not generally observed in plant systems. Resilience, on the other hand, is the ability to survive these changes despite severe impact, which is more desirable and what is generally observed in plant systems. Both systems (i.e., with and without the complex) are robust and achieves robustness by redundancy (multiple paths towards S and G). However, the system that includes the development of a Ptr4CL3-Ptr4CL5 complex improves the efficiency of the Ptr4CL enzymes and their ability to use the substrates or switch between substrates more effectively [9].

In the presence of the Ptr4CL3-Ptr4CL5 complex, the variation in the steady state flux V_7_ can be visualized in Fig 5. Under WT Ptr4CL3 and Ptr4CL5 concentrations, the steady state flux is at its maximum value of 0.7 μM/min. As seen in the Fig 5, the change in flux V_7_ is primarily due to the changes in Ptr4CL5 concentration. When the concentration of Ptr4CL5 is reduced by 50% of its WT concentration while holding the concentration of Ptr4CL3 at its WT level, we observe a 10% reduction in the steady state flux. To observe a similar 10% change in the steady state flux, the concentration of Ptr4CL3 would need to be reduced by 80% of its WT, with only a 20% reduction in the Ptr4CL5 concentration. These observations confirm that smaller adjustments in the Ptr4CL3 and Ptr4CL5 can result in significant change in the conversion rate of p-coumaric acid to 4-coumaroyl-CoA when the Ptr4CL3-Ptr4CL5 complex is formed. The presence of the complex provides the plant with more tuneability in flux distribution among V_7_-V_9_. Moreover, from a biological perspective, the plant has developed an efficient regulatory control scheme. If the plant were to regulate 4CL activity by synthesizing and/or degrading 4CL3 enzymes, then it would be highly energy-intensive and slow since 4CL3 protein must be highly abundant according to the results in Fig 4. Therefore, by complexing a less abundant 4CL5 to 4CL3, plants can more efficiently regulate the overall 4CL activity by simply adjusting the abundance of the 4CL5 enzyme (Fig 5).

The variations in all the metabolic fluxes involved in the biosynthesis are summarized in S7 Fig. In the presence of a complex, the variation of Ptr4CL concentration results in a large variability of the steady state flux (denoted by either wider spreads in the flux distributions or the presence of bimodal distributions). The variation is primarily due to the changes in concentrations of Ptr4CL3-Ptr4CL5 complex. The metabolic flux model with the complex is significantly more sensitive to the concentrations of Ptr4CL5 enzyme that leads to large variability in the steady state flux. Such increases in the variance of the individual fluxes in the pathway allows for more efficient regulatory control from lower concentrations of Ptr4CL5 enzyme to achieve a desired lignin content and structure.

### Role of Ptr4CL complex on S and G monolignols, lignin content and composition

So far, we discussed the prediction of the steady state metabolite and flux distributions for the model with and without the Ptr4CL3-Ptr4CL5 complex and explored the impact of varying the Ptr4CL3 and Ptr4CL5 concentrations. In this section, we extend the discussion to the total lignin content and S/G ratio. Down-regulation of the Ptr4CL activity results in decreased lignin content in alfalfa, arabidopsis, tobacco, aspen (*Populus tremuloides*) and hybrid white poplar (*Populus tremula X Populus alba*) [25,26]. This decrease in lignin content results in a slight increase in the S/G ratio in alfalfa, a greater increase in S/G ratio in Arabidopsis, an unchanged S/G ratio in aspen, and a decreased S/G ratio in hybrid white poplar [27]. The reduction in lignin content in Arabidopsis following down-regulation of Ptr4CL is achieved by a decrease in G units but not S units, leading to a higher S/G ratio and the plants appearing phenotypically normal [14]. Researchers have shown that a down regulation of Ptr4CL enzyme in poplar results in a decrease in total lignin content and no change in S/G ratio [13,28]. In the absence of a complex and for a wide range of Ptr4CL concentrations, the model’s S and G subunits do not change from the WT level. At very low concentrations of Ptr4CL, the model’s S and G subunits approach zero (Fig 4). In the presence of a complex and at higher concentrations of Ptr4CL, lignin is primarily composed of S and G monolignols; but as the concentration of Ptr4CL is reduced, the model predicted a significant reduction in lignin content (Fig 5). This result is in agreement with the results observed in the literature [13,28]. From the above results, we can conclude that the inclusion of the Ptr4CL complex in the model provides us with a more comprehensive representation of the regulation of lignin content and composition in *P*. *trichocarpa*, when the pathway is subjected to Ptr4CL family perturbations.

The changes in Ptr4CL3 and Ptr4CL5 did not affect the total lignin content and lignin composition for the model in the absence of a complex. This lack of impact on lignin content and lignin composition was expected since the changes in the concentrations of Ptr4CL3 and Ptr4CL5 did not significantly affect the steady state distribution of the metabolic flux (Fig 2). However, the model with the complex does display some deviation from the wildtype, leading to a 20 percent reduction in lignin content that does not have wildtype properties. The variation of S and G monolignol units when the Ptr4CL3 and Ptr4CL5 concentrations are perturbed in the presence of the complex is shown in S8 Fig. About 80% of the S and G units correspond to the WT levels, while at low concentrations of Ptr4CL, the S and G units are reduced.

The effect of varying Ptr4CL3 and Ptr4CL5 levels on the S/G ratio in the absence of a complex can be seen in the contour plot shown in S9 Fig. As seen in S9 Fig., changes in the Ptr4CL concentration do not result in a significant change in the S/G ratio over a wide range of values. When the Ptr4CL3 concentration was reduced to less than 10% of its WT concentration, the S/G ratio increased from its WT ratio. However, these results are not biologically achievable because these reductions in Ptr4CL concentrations would result in plants with very low lignin content, a condition that would produce plants lacking mechanical strength [25,29].

The variation in S/G ratio for the model with Ptr4CL3-Ptr4CL5 complex as a function of the Ptr4CL3 and Ptr4CL5 concentrations is shown in S10 Fig. These results confirm the regulatory role of Ptr4CL5 in the presence of the Ptr4CL3-Ptr4CL5 complex [9,11]. The contour plot suggests that for the model with the complex, changes in S/G ratio and potentially the lignin structure is primarily due to variation in Ptr4CL5. The S/G ratio is robust to perturbations until the concentration of Ptr4CL5 is reduced to 60% of the WT concentration; any further change in Ptr4CL5 levels results in a linear change in S/G ratio.

From the above results, the variation in lignin content and composition as a function of changes in concentrations of Ptr4CL is significantly influenced by the presence of the Ptr4CL3-Ptr4CL5 complex. Hence, the inclusion of the complex into the model enhances our understanding about the regulation of metabolic flux through the pathway. These results are consistent with experimental results from the literature, which suggest that the lignin content and structure can be altered by targeting specific genes in the monolignol biosynthetic pathway. These results confirm that the presence of complex in the Ptr4CL protein family is a critical component in the regulation of the pathway. The role of multi-enzyme complex on the genetic manipulation of lignin was previously studied by [30] Campbell and Sederoff (1996). However, the exact role of the complex was not evident at that time. The simulation results of the predictive kinetic metabolic flux (PKMF) model [8] suggest that there is an opportunity to genetically modify the lignin content and structure by targeting the Ptr4CL5 enzyme. Although the role of the Ptr4CL3-Ptr4CL5 complex on the lignin content and structure were identified, the effect of changing levels of Ptr4CL3 and/or Ptr4CL5 enzyme concentrations on the individual reaction rates can be quantified by performing a sensitivity analysis provided in the next section.

### Sensitivity analysis of Ptr4CLs on the monolignol biosynthetic pathway

**Fig 6:**
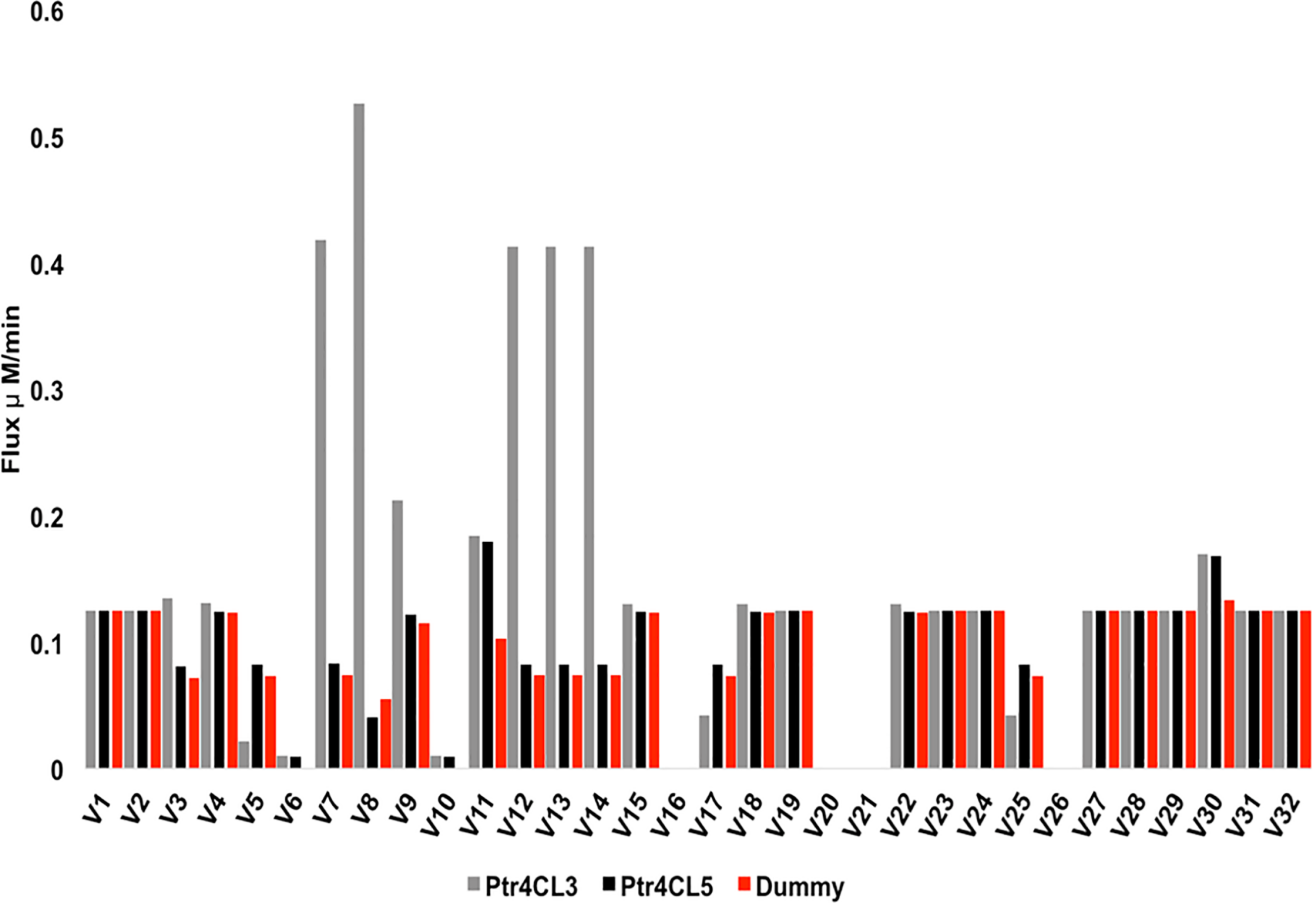
The first order sensitivity index for monolignol flux with respect to Ptr4CL3 and Ptr4CL5 concentrations for the model without the complex.

**Fig 7:**
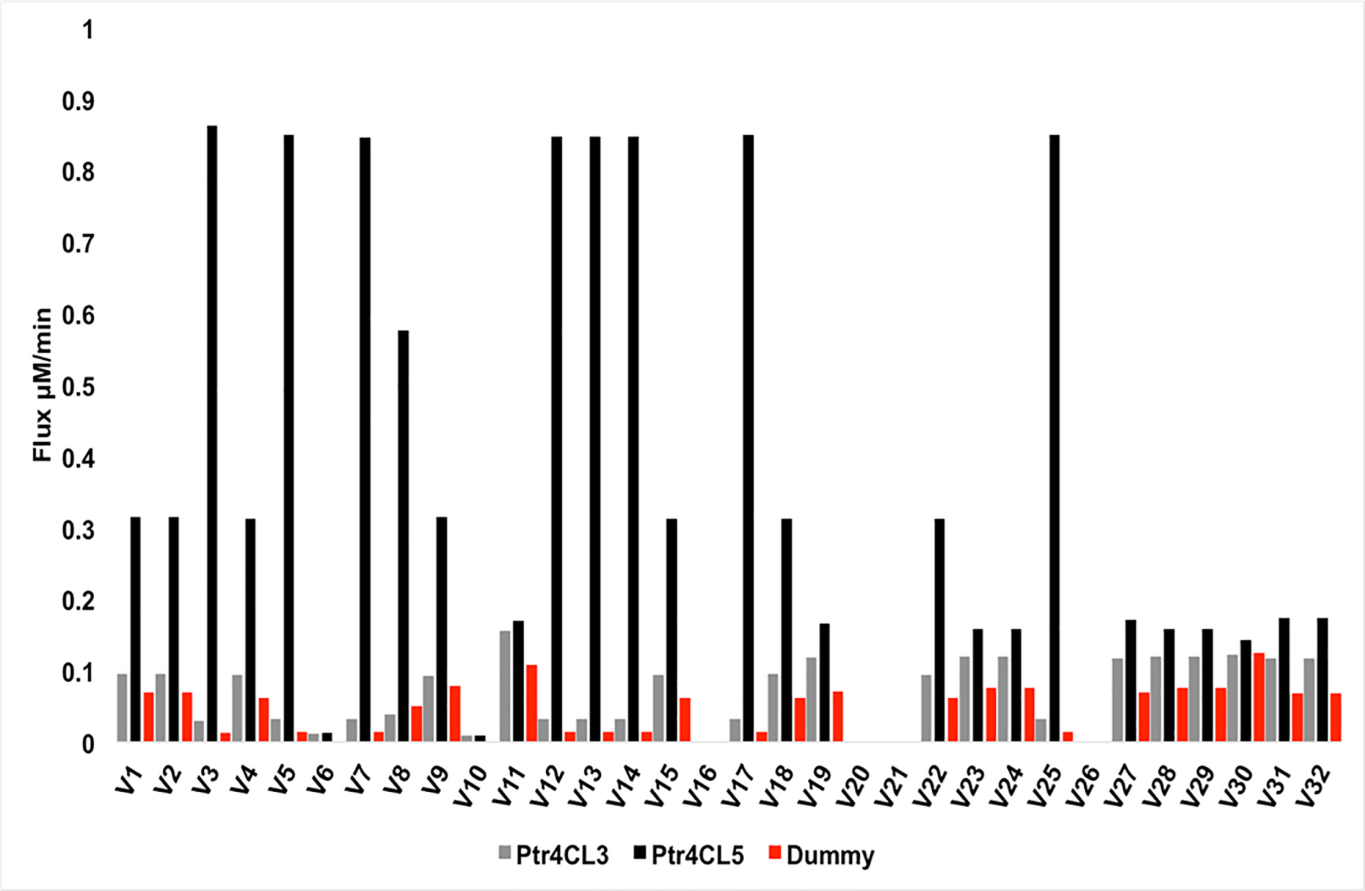
The first order sensitivity index for monolignol flux with respect to Ptr4CL3 and Ptr4CL5 concentrations for the model with complex.

Since the variation of the reaction flux with Ptr4CL enzymes were non-monotonic, we used the variance decomposition method [31] to assess the sensitivity of steady state reaction flux to Ptr4CL concentration. The concentrations of other enzymes were fixed at their WT levels. The sensitivity of the steady state flux to changes in the concentrations of Ptr4CL3 and Ptr4CL5 enzymes in the absence and presence of the Ptr4CL3-Ptr4CL5 complex is shown in Fig 6 and Fig 7, respectively. The first order sensitivity index measures the percentage of variance explained by varying the enzymes independently. To quantify the role of individual enzymes on the resulting flux or substrate concentration, the first order sensitivity indices for Ptr4CL3 and Ptr4CL5 were compared against a dummy parameter, which is an arbitrary parameter that has no influence on the model [32]. The dummy parameter has no effect on the model as it does not appear in any of the equations and hence should have a very low sensitivity. The sensitivity index for Ptr4CL3 and Ptr4CL5 that is significantly different from the dummy parameter indicates that the particular enzyme has an influence on the resulting flux or substrate concentration. In Fig 6, the reaction flux is primarily affected by changes in the levels of Ptr4CL3 in the absence of a complex. The fluxes that are affected mainly involve the reactions mediated by Ptr4CL enzymes and the reactions near the Ptr4CL pathway.

In the presence of a Ptr4CL3-Ptr4CL5 complex, the reactions mediated by Ptr4CLs (V_7_, V_8_ and V_9_) are especially sensitive to the changes in Ptr4CL5 (Fig 7). The terminal reactions towards the end of the biosynthetic pathway, which are directly responsible for the lignin content and structure (V_27_, V_29_, V_31_, and V_32_) all show a slightly higher sensitivity to variations in Ptr4CL3 and Ptr4CL5 concentrations for the model with the complex. The changes in the terminal metabolic flux might indicate the reason as to why we observe a distribution of S/G ratios around the WT Ptr4CL concentrations (see S10 Fig).

### Robustness of monolignol biosynthetic pathway

Robustness, a property that allows a system to maintain its functions in the presence of large environmental perturbations [33,34], is a widely discussed topic in systems biology [33,35]. The primary function in our case represents maintaining the levels of S/G ratio and the total lignin content (S+G). The biosynthesis of monolignols is a metabolic grid, which means the existence of multiple routes towards the synthesis of monolignol subunits (S1 Fig). This potential flexibility in synthesis routes gives plants the ability to maintain near wild type lignin structure and composition when a step is inhibited. In the presence of a complex and depending on the level of Ptr4CL perturbation, the flux through the monolignol pathway can be routed through p-coumaric acid (V_7_) or caffeic acid (V_8_) (S3 Fig). In cases of extreme perturbation, plants are known to have incorporated other phenolic components into the monolignol subunits [36]. As seen for the case of the model with the complex, the changes in Ptr4CL levels resulted in a proportional change in the S and G monolignol levels, with a majority of S and G monolignol subunits distributed around their WT concentrations. This result suggest that the presence of a Ptr4CL complex leads to increased plasticity of the pathway.

Robustness applies to a few variables or a particular part of the model and not necessarily for the entire system; a key difference from stability where it is a property of the entire system. It has been suggested that there is some form of equilibrium in robustness where some part of the pathway may be sensitive to perturbations while the remaining pathway remains unaffected [35]. A similar scenario was observed in our case, where the reaction flux corresponding to the early steps of the monolignol biosynthesis pathways were more sensitive to the changes in the levels of Ptr4CL3 and Ptr4CL5 concentrations. As we move downstream towards the terminal reactions, the sensitivity of those reactions was considerably reduced. From our sensitivity analysis, the pathway is fairly robust to changes in levels of Ptr4CL3 and Ptr4CL5 concentrations under mild perturbations for the pathway that does not contain the enzyme complex.

The dynamic properties of most networks would also lead to a change in their robustness [37]. Therefore, dynamic networks can continue to be simultaneously robust and elastic. We observed a similar property in the model containing the complex where the pathway is able to maintain a continuous production of lignin despite perturbations to the levels of Ptr4CL3 and Ptr4CL5. The variation in lignin content and composition as a function of changes in concentrations of Ptr4CL is significantly influenced by the presence of the Ptr4CL3-Ptr4CL5 complex. In the absence of complex, the model suggests that the S/G ratio remains 2:1 irrespective of these perturbations, which is not consistent with the experimental results from the literature [30]. These results explain the role of the complex as well as provide insights into why plants may produce enzyme complexes.

### Stability analysis

**Fig 8:**
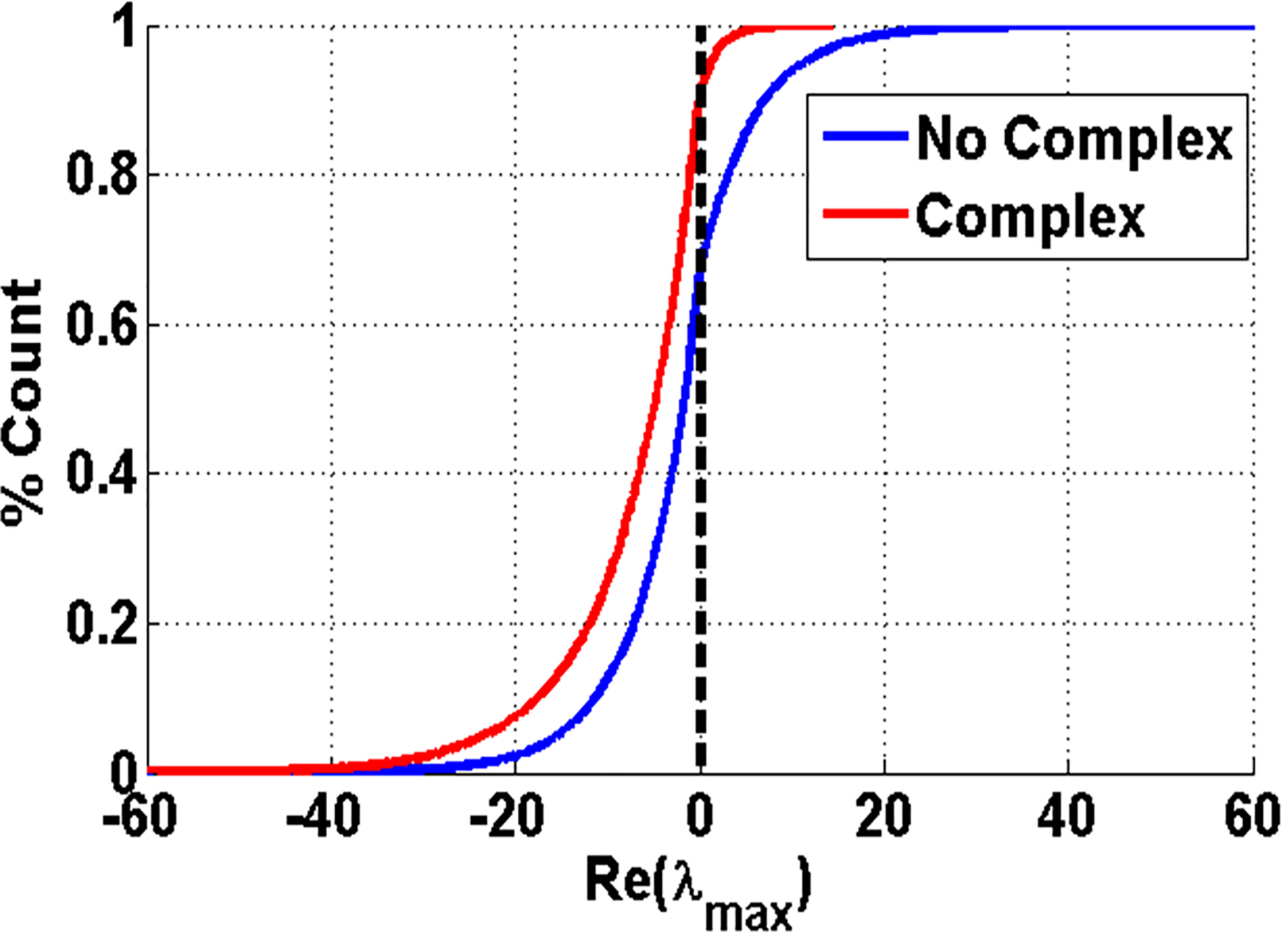
Cumulative distribution function plot of Eigenvalues for the model with and without the complex under WT enzyme concentrations.

The role of Ptr4CL3-Ptr4CL5 complex on the overall monolignol biosynthetic pathway can also be assessed by performing a stability analysis. Since the presence of the complex affects the steady state flux distribution through feed forward and feedback inhibition, the complex affects the dynamic properties of the biosynthetic pathway. The Jacobian matrix was evaluated for models with and without the complex based on the i^th^ λ_Re_^max^ > 0 implying instability of the metabolic state [21]. A set of 10,000 model evaluations were performed for different steady state metabolite concentrations resulting from different enzyme concentrations of Ptr4CL3 and Ptr4CL5 for the model with and without the Ptr4CL3-Ptr4CL5 complex. Based on the sign of the Eigenvalue for each set of metabolite and enzyme concentrations, the model was classified into stable or unstable conditions. The resulting Eigenvalues were then plotted as a histogram of Eigenvalues for these 10,000 iterations. The cumulative distribution function (CDF) of the Eigen value distribution is shown in Fig 8 for the case of model with and without the complex. From Fig 8, the lignin biosynthetic model is inherently stable for both cases, which suggests that under perturbations, the system is robust to small changes in enzyme levels. These results support the experimental findings that the S/G ratio of lignin in most transgenic plants is around the WT S/G ratio. The presence of the Ptr4CL3-Ptr4CL5 complex, however, increased the percentage of model stable conditions from 70% to 92%. The percentage of stable models were calculated by counting the number of models with negative eigenvalues (ƛ_max_) divided by the total number of models (10,000). Although a large percentage of the models are stable, a small percentage of unstable models cannot be neglected, as these steady states indicate that the network could be driven out of the observed steady state when Ptr4CL3 and more specifically Ptr4CL5 concentrations are perturbed based on our analyses. The increased stability in the model containing the enzyme complex enables plants to maintain the lignin composition and structure under normal external or internal perturbations.

The results from stability analysis suggests that although the efficiency of the Ptr4CL enzymes increase when they form a complex, the primary reason for the complex formation may be because they are able to help the plant maintain homeostasis more efficiently. This claim is supported by the results from the model that, in the presence of the Ptr4CL3-Ptr4CL5 complex, the pathway is able to use both p*-*coumaric acid and caffeic acid as substrates to produce lignin. The results of varying the enzyme concentrations suggest that the monolignol biosynthetic pathway is homeostatic in both the absence and presence of the complex. The flux distribution for the model without the complex appears to be unaffected with the variation in Ptr4CL concentrations. However in the presence of a complex, the model exhibits multi-stability; i.e., the steady state flux corresponding to high enzyme concentrations are similar to the steady state flux observed for the model without the complex. At very low concentrations of Ptr4CL, however, the model results in an additional steady state.

## Conclusions

The Monte Carlo simulations of the PKMF model enables us to identify all the feasible steady state concentrations of the metabolites involved in the pathway. This approach provides a comprehensive analysis of the entire monolignol biosynthesis pathway involving all the metabolites, enzymes, and the multi-enzyme complex such as Ptr4CL3-Ptr4CL5 complex. The results from this analysis enabled us to quantify the effect of perturbing the Ptr4CL enzymes on the steady state flux distribution within the pathway as well as its effect on the lignin content and structure. In the presence of perturbing the Ptr4CL enzymes, the simulation results suggest that the presence of Ptr4CL3-Ptr4CL5 complex produces a redundant pathway towards the biosynthesis of S and G monolignols. In addition, the Ptr4CL3-Ptr4CL5 complex enhances the robustness of the pathway and leads to the stability of the metabolic states of the different metabolites involved in the pathway. The local stability analysis suggests that the presence of Ptr4CL3-Ptr4CL5 complex increases the stability of the metabolic network by 22%.

Moreover, the perturbation of the Ptr4CL enzymes with the Ptr4CL3-Ptr4CL5 complex results in a bimodal distribution of steady state flux. This bimodal distribution is primarily due to the feedback inhibition that exists between the various hydroxy cinnamic acids involved in the Prt4CL pathway. The analysis of the PKMF model also suggests that the Ptr4LC5 enzyme plays a key regulatory role in the modulation of the metabolic flux. These results further confirm the experimental findings that Ptr4CL5 is essential for regulating the levels of caffeic acid that is essential to moderate the CoA flux ligation [11]. The sensitivity analysis performed on the PKMF model suggests that the perturbation of Ptr4CL3 and Ptr4CL5 enzymes results in only a localized change in the steady state metabolic flux in the absence of a complex. However, in the presence of the complex, the steady state flux was sensitive to changes in Ptr4CL5 concentrations, further confirming the regulatory role of Ptr4CL5 enzyme.

## Supporting information

S1 Fig The monolignol biosynthetic pathway in *P. trichocarpa*. Thirty-five metabolic fluxes (V0 to V35, represented by the circled numbers) mediate the conversion of 24 metabolites (underlined numbers) for monolignol synthesis by the 21 pathway enzymes (Wang et al., 2014).
(TIF)

S2 Fig Steady state flux (V_7_, V_8_ and V_9_) variation as a function of total Ptr4CL concentration for models without the Ptr4CL3-Ptr4CL5 complex. (TIF)

S3 Fig Steady state flux (V7, V8 and V9) variation as a function of total Ptr4CL concentration for models with the Ptr4CL3-Ptr4CL5 complex. (TIF)

S4 Fig Ptr4CL pathway inhibitory reactions. (TIF)

S5 Fig Violin plot showing the steady state flux (V_7_, V_8_ and V_9_) variation as a function of total Ptr4CL concentration for models in the presence of the Ptr4CL3-Ptr4CL5 complex. (TIF)

S6 Fig Variation in the distributions of P-coumaric (μM), caffeic (μM), and ferulic (μM) acid concentrations in the presence of the Ptr4CL3-Ptr4CL5 complex. (TIF)

S7 Fig The steady state distribution of all the metabolic flux involved in the monolignol biosynthetic pathway. (a) The steady state flux distribution in the absence of Ptr4CL3-Ptr4CL5 complex. The green box represents the median steady state flux and the red ‘+’ sign represents the mean steady state flux values, (b) The steady state flux distribution in the presence of Ptr4CL3-Ptr4CL5 complex. The presence of complex induces a bimodal steady state flux distributions. The green box represents the median steady state flux and the red ‘+’ sign represents the mean steady state flux values.
(TIF)

S8 Fig Variation of monolignol units resulting due to changes in levels of Ptr4CL3 and Ptr4CL5 concentrations in the presence of a complex: a) S and b) G.
(TIF)

S9 Fig Variation of S/G ratio as a function of Ptr4CL3 and Ptr4CL5 concentration in the absence of a complex. The color bar shows the variation of S/G ratio in log scale, where S/G ratio of 2 corresponds to a value of 0.3 in log scale.
(TIF)

S10 Fig Variation of S/G ratio as a function of Ptr4CL3 and Ptr4CL5 concentration in the presence of a complex. The color bar shows the variation of S/G ratio in log scale.
(TIF)

S1 Table List of all the reactions involved in the monolignol biosynthesis pathway. (DOCX)

S2 Table List of all flux kinetic equations in the monolignol biosynthesis pathway. (PDF)

S3 Table Description of flux equations for Ptr4CL enzyme related pathways with and without the complex. (PDF)

## References

[1] Sarkanen KV and Ludwig CH. Lignin: Occurrence, Formation, Structure and Reactions. C.H. Wiley-Interscience: New York 1971.

[2] Higuchi T.Biochemistry and Molecular Biology of Wood. Springer-Verlag, Berlin-Heidelberg-New York 1997.

[3] Chiang VL From rags to riches. Nat Biotechnol 2002; 20: 557–558. doi: 10.1038/nbt0602-557 12042854 10.1038/nbt0602-557

[4] Ragauskas AJ, Williams CK, Davison BH, Britovsek G, Cairney J, Eckert CA, et al The Path Forward for Biofuels and Biomaterials. Science 2006; 311:484–489. doi: 10.1126/science.1114736 16439654 10.1126/science.1114736

[5] Lee Y, Chen F, Gallego-Giraldo L, Dixon RA, Voit EO. Integrative analysis of transgenic alfalfa (Medicago sativa L.) suggests new metabolic control mechanisms for monolignol biosynthesis. PLOS Comput. Biol. 2011; 7(5): e1002047 doi: 10.1371/journal.pcbi.1002047 21625579 10.1371/journal.pcbi.1002047 PMC3098223

[6] Lee Y, Voit EO. Mathematical modeling of monolignol biosynthesis in Populus xylem. Math. Biosci. 2010; 228: 78–89. doi: 10.1016/j.mbs.2010.08.009 20816867 10.1016/j.mbs.2010.08.009

[7] Schallau K, Junker BH. Simulating plant metabolic pathways with enzyme-kinetic models. Plant Physiol. 2010; 152: 1763–1771. doi: 10.1104/pp.109.149237 20118273 10.1104/pp.109.149237 PMC2850014

[8] Wang JP, Naik PP, Chen HS, Shi R, Lin CY, Liu J, et al Complete Proteomic-Based Enzyme Reaction and Inhibition Kinetics Reveal How Monolignol Biosynthetic Enzyme Families Affect Metabolic Flux and Lignin in *Populus trichocarpa*. Plant Cell, 2014; 26 (3), 894–914. doi: 10.1105/tpc.113.120881 24619611 10.1105/tpc.113.120881 PMC4001400

[9] Chen H., Song J., Wang J.P., Lin Y., Ducoste J., Shuford C.M., et al Systems Biology of Lignin Biosynthesis in *Populus trichocarpa*: Heteromeric 4-Coumaric Acid:Coenzyme A Ligase Protein Complex Formation, Regulation, and Numerical Modeling, Plant Cell, 2014; http://dx.doi.org/10.1105/tpc.113.119685 10.1105/tpc.113.119685 PMC4001399 24619612

[10] Marsh J.A., Rees H.A., Ahnert S.E., Teichmann S.A. Structural and evolutionary versatility in protein complexes with uneven stoichiometry, Nat. Commun. 2015; 6:6394 doi: 10.1038/ncomms7394 25775164 10.1038/ncomms7394

[11] Chen H.C., Song J., Williams C.M., Shuford C.M., Liu J., Wang J.P., et al Monolignol Pathway 4-Coumaric Acid:Coenzyme A Ligases in *Populus trichocarpa*: Novel Specificity, Metabolic Regulation, and Simulation of Coenzyme A Ligation Fluxes, Plant Physiology, 2013; Vol. 161, pp. 1501–1516 doi: 10.1104/pp.112.210971 23344904 10.1104/pp.112.210971 PMC3585612

[12] Kajita S, Hishiyama S, Tomimura Y, Katayama Y, Omori S. Structural characterization of modified lignin in transgenic tobacco plants in which the activity of 4-coumarate: coenzyme A ligase is depressed. Plant Physiology 1997; 114: 871–879. 12223748 10.1104/pp.114.3.871 PMC158374

[13] Hu W., Harding S.A., Lung J., Popko J.L., Ralph J., Stokke D.D., et al Repression of lignin biosynthesis promotes cellulose accumulation and growth in transgenic trees, Nature Biotechnology 1999; 17, 808–812 doi: 10.1038/11758 10429249 10.1038/11758

[14] Lee D., Meyer K., Chapple C., and Douglas C.J. Antisense suppression of 4-coumarate:coenzyme A ligase activity in Arabidopsis leads to altered lignin subunit composition. Plant Cell 1997; 9:1985–1998. doi: 10.1105/tpc.9.11.1985 9401123 10.1105/tpc.9.11.1985 PMC157052

[15] Kajita S., Katayama Y., and Omori S. Alterations in the biosynthesis of lignin in transgenic plants with chimeric genes for 4- coumarate: coenzyme A ligase. Plant Cell Physiol. 1996; 37: 957–965. 8979396 10.1093/oxfordjournals.pcp.a029045

[16] Helton JC, Iman RL and Brown JB. Sensitivity Analysis of the Asymptotic Behavior of a Model for the Environmental Movement of Radionuclides, Ecol. Modelling. 1985; 28, 243–278.

[17] Sumner T., Shephard E., Bogle I. D. L. A methodology for global-sensitivity analysis of time-dependent outputs in systems biology modelling. *Journal of the Royal Society Interface*. 2012; 9(74):2156–2166. doi: 10.1098/rsif.2011.0891 10.1098/rsif.2011.0891 PMC3405739 22491976

[18] Marino S., Hogue I.B., Ray C.J., Kirschner D.E., A Methodology For Performing Global Uncertainty And Sensitivity Analysis In Systems Biology J Theor Biol. 2008; 254(1): 178–196.p doi: 10.1016/j.jtbi.2008.04.011 18572196 10.1016/j.jtbi.2008.04.011 PMC2570191

[19] Strogatz S.H. Nonlinear Dynamics and Chaos, Westview Press, Perseus Books Publishing 1994.

[20] Kelley CT. Solving nonlinear equations with Newton`s method. SIAM, xiv/ISBN: 978-0-898715-46-0, 2003.

[21] Steuer R, Waldherr S, Sourjik V, Kollmann M. Robust Signal Processing in Living Cells. PLoS Comput Biol 2011; 7(11): e1002218 doi: 10.1371/journal.pcbi.1002218 22215991 10.1371/journal.pcbi.1002218 PMC3219616

[22] Grimbs S., Selbig J., Bulik S., Holzhütter H.-G. & Steuer R. The stability and robustness of metabolic states: identifying stabilizing sites in metabolic networks. Mol Syst Biol 2007; 3, 146 doi: 10.1038/msb4100186 18004279 10.1038/msb4100186 PMC2132447

[23] Girbig D., Grimbs S., Selbig J. Systematic analysis of stability patterns in plant primary metabolism. PLoS One, 2012; 7(4), e34686 doi: 10.1371/journal.pone.0034686 22514655 10.1371/journal.pone.0034686 PMC3326025

[24] Shi R, Sun YH, Li Q, Heber S, Sederoff R. and Chiang VL. Towards a systems approach for lignin biosynthesis in Populus trichocarpa: transcript abundance and specificity of the monolignol biosynthetic genes. Plant Cell Physiol. 2010; 51, 144–163. doi: 10.1093/pcp/pcp175 19996151 10.1093/pcp/pcp175

[25] Voelker S.L., Lachenbruch B., Meinzer F.C., Jourdes M., Ki C., Patten A.M., et al Antisense down-regulation of 4CL expression alters lignification, tree growth, and saccharification potential of field-grown poplar. Plant Physiology 2010; 154: 874–886. doi: 10.1104/pp.110.159269 20729393 10.1104/pp.110.159269 PMC2949011

[26] Kitin P., Voelker S.L., Meinzer F.C., Beeckman H., Strauss S.H., and Lachenbruch B. Tyloses and phenolic deposits in xylem vessels impede water transport in low-lignin transgenic poplars: A study by cryo-fluorescence microscopy. Plant Physiol. 2010; 154: 887–898. doi: 10.1104/pp.110.156224 20639405 10.1104/pp.110.156224 PMC2949004

[27] Romani A Lattanzio V and Quideau S. Recent advances in polyphenol research. ISBN: 9781118329634. 2014.

[28] Li L., Zhou Y., Cheng X., Sun J., Marita J.M., Ralph J., et al Combinatorial modification of multiple lignin traits in trees through multigene cotransformation, PNAS, 2003; vol. 100 no. 8, 4939–4944, doi: 10.1073/pnas.0831166100 12668766 10.1073/pnas.0831166100 PMC153659

[29] Coleman H.D., Samuels A.L., Guy R.D., Mansfield S.D. Perturbed lignification impacts tree growth in hybrid poplar–a function of sink strength, vascular integrity, and photosynthetic assimilation. Plant Physiology 2008; 148: 1229–1237. doi: 10.1104/pp.108.125500 18805953 10.1104/pp.108.125500 PMC2577275

[30] Campbell MM and Sederoff RR. (1996) Variation in Lignin Content and Composition (Mechanisms of control and implications for the genetic improvement of plants). Plant Physiology, 110:3–13. 12226169 10.1104/pp.110.1.3 PMC157688

[31] Sobol I. Sensitivity analysis for non-linear mathematical models. Mathematical Modeling & Computational Experiment, 1993; 1, 407–414.

[32] Saltelli A, Chan K, Scott EM. Wiley series in probability and statistics Wiley, Chichester; New York: Sensitivity analysis. 2000.

[33] Kitano H. Biological robustness. Nat Rev Genet, 2004; 5 826–837 doi: 10.1038/nrg1471 15520792 10.1038/nrg1471

[34] Kitano H. Towards a theory of biological robustness. Mol Syst Biol, 2007; 3: 137 doi: 10.1038/msb4100179 17882156 10.1038/msb4100179 PMC2013924

[35] Zhou K., Doyle J.C., and Glover K. Robust and Optimal Control (Upper Saddle River: Prentice Hall). 1995.

[36] Ralph J, Lundquist K, Brunow G, Lu F, Kim H, Schatz PF et al Lignins: natural polymers from oxidative coupling of 4-hydroxyphenyl-propanoids. Phytochem. Rev. 2004; 3, 29–60.

[37] Matisziw TC, Grubesic TH, Guo J. Robustness Elasticity in Complex Networks. PLoS ONE, 2012; 7(7): e39788 doi: 10.1371/journal.pone.0039788 22808060 10.1371/journal.pone.0039788 PMC3393721

