# Supplementary figures and images for: Assessing the impact of the 4CL enzyme complex on the robustness of monolignol biosynthesis using metabolic pathway analysis
Source: PLoS One. 2018 Mar 6;13(3):e0193896. doi: 10.1371/journal.pone.0193896 (PMC5839572; doi:10.1371/journal.pone.0193896)

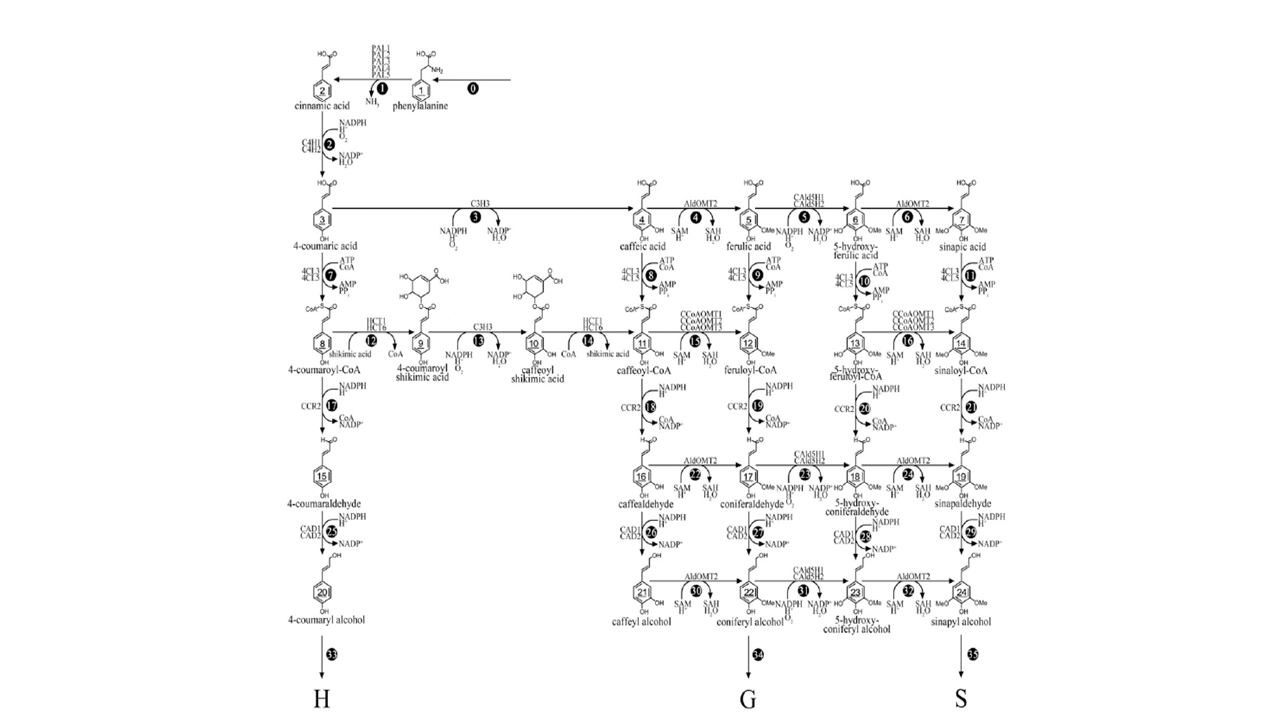

Supplement: S1 Fig — Thirty-five metabolic fluxes (V0 to V35, represented by the circled numbers) mediate the conversion of 24 metabolites (underlined numbers) for monolignol synthesis by the 21 pathway enzymes (Wang et al., 2014). (TIF) [file pone.0193896.s001.tif]

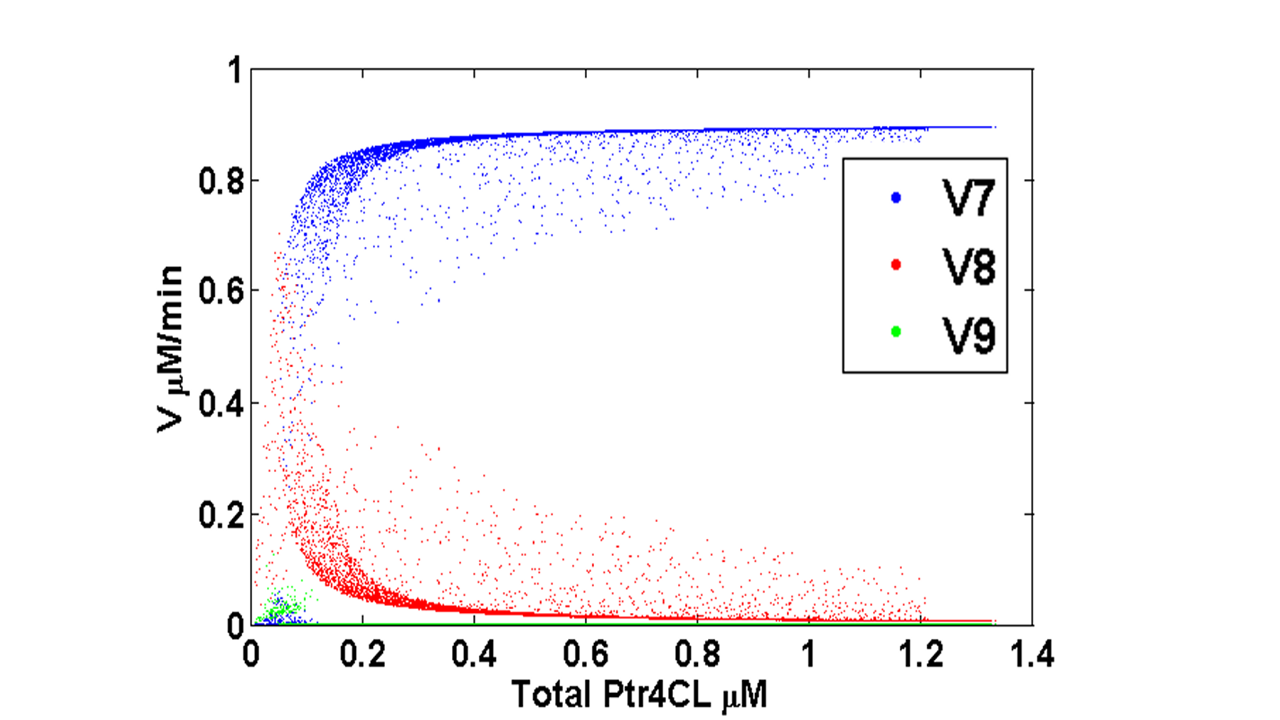

Supplement: S2 Fig — (TIF) [file pone.0193896.s002.tif]

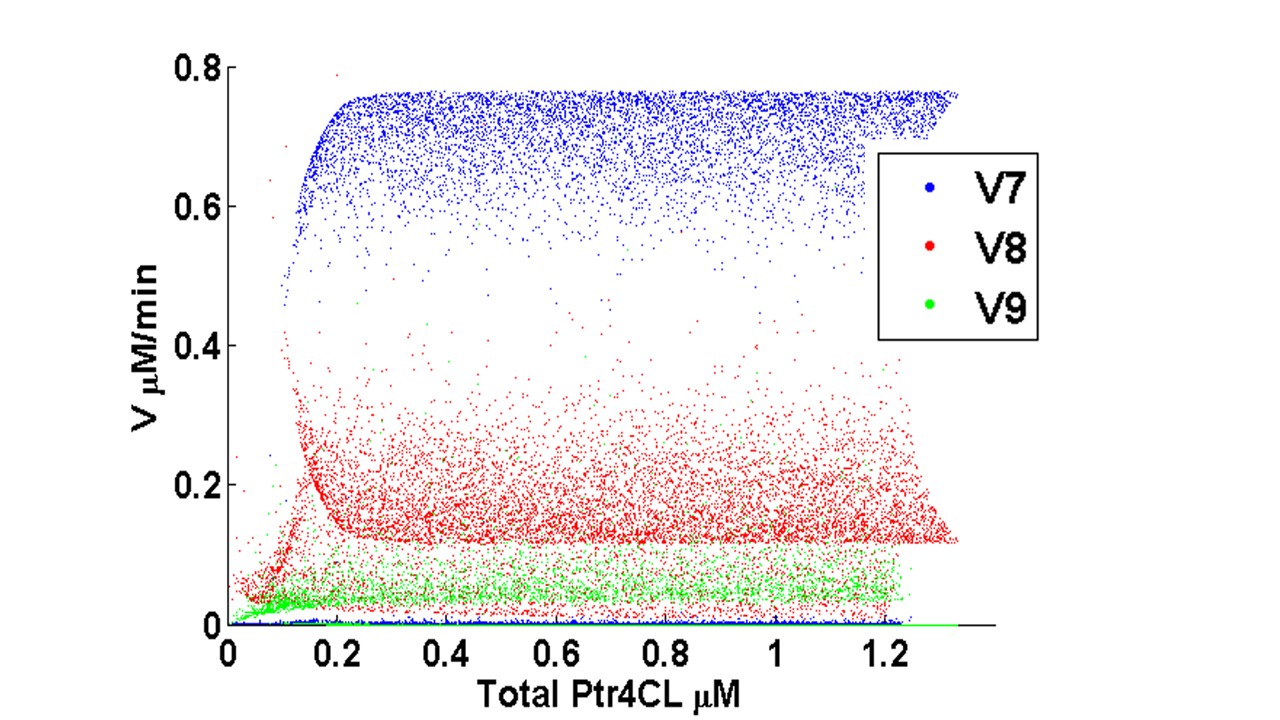

Supplement: S3 Fig — (TIF) [file pone.0193896.s003.tif]

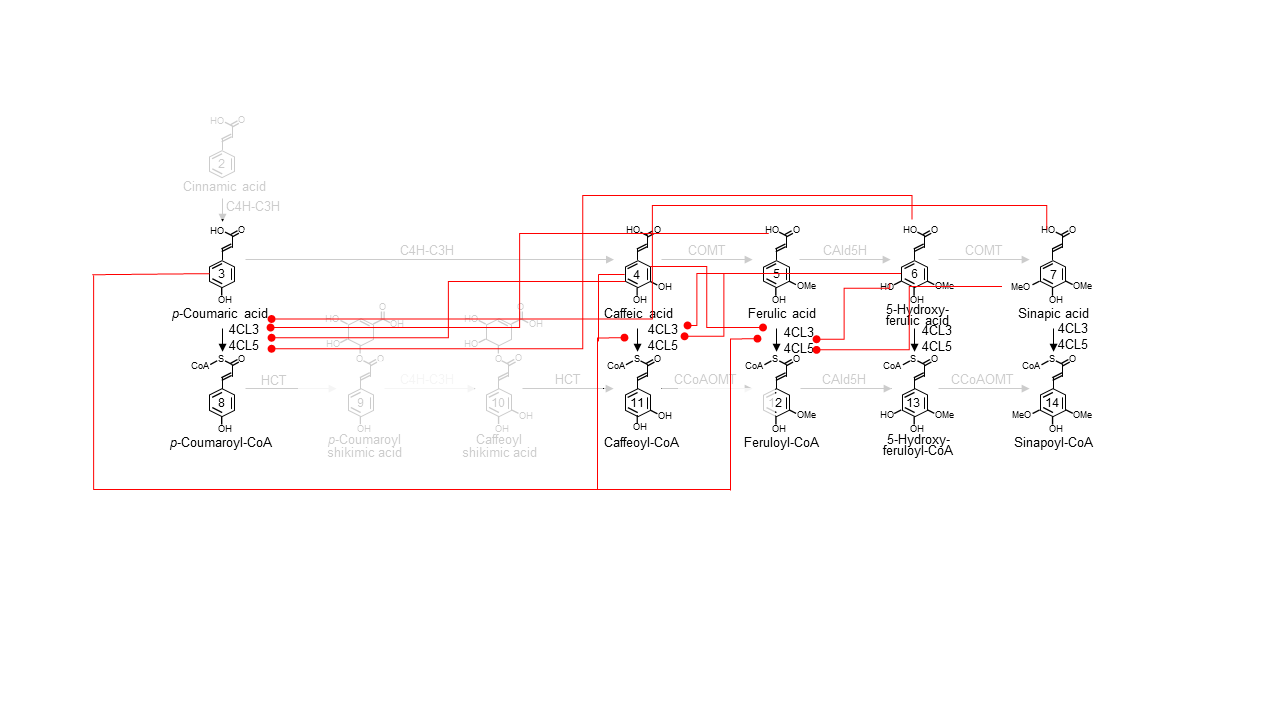

Supplement: S4 Fig — (TIF) [file pone.0193896.s004.tif]

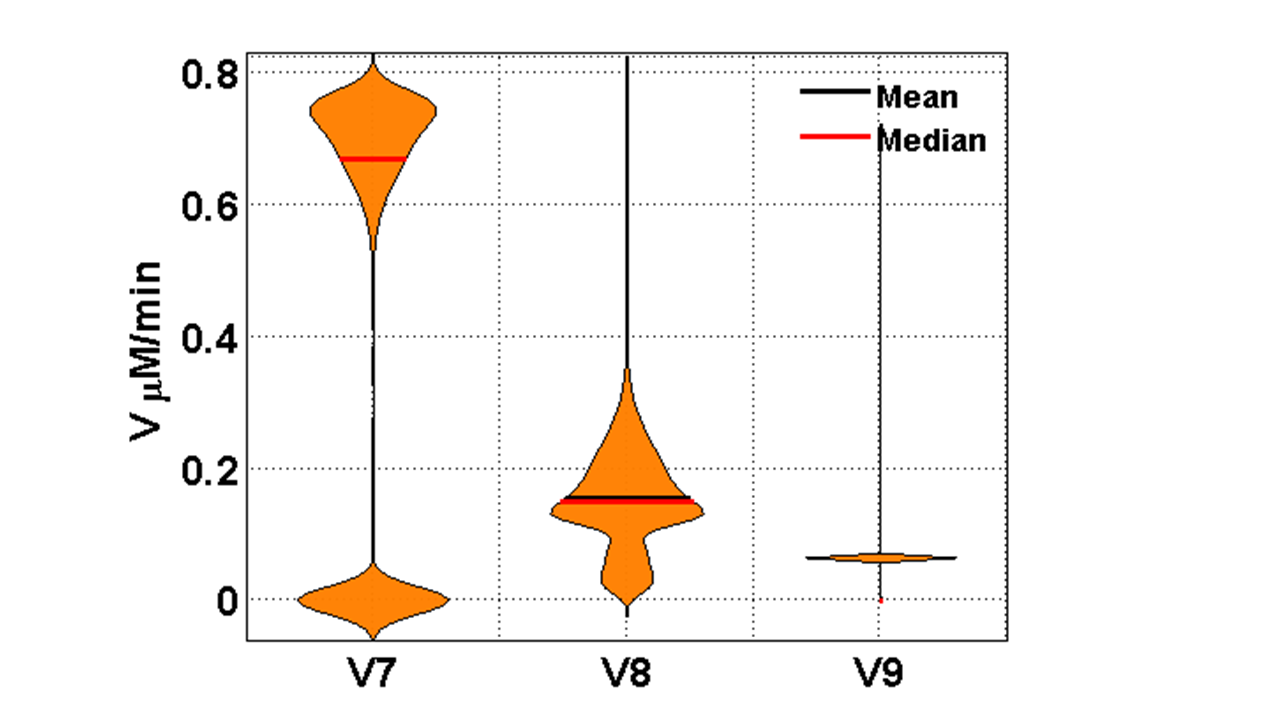

Supplement: S5 Fig — (TIF) [file pone.0193896.s005.tif]

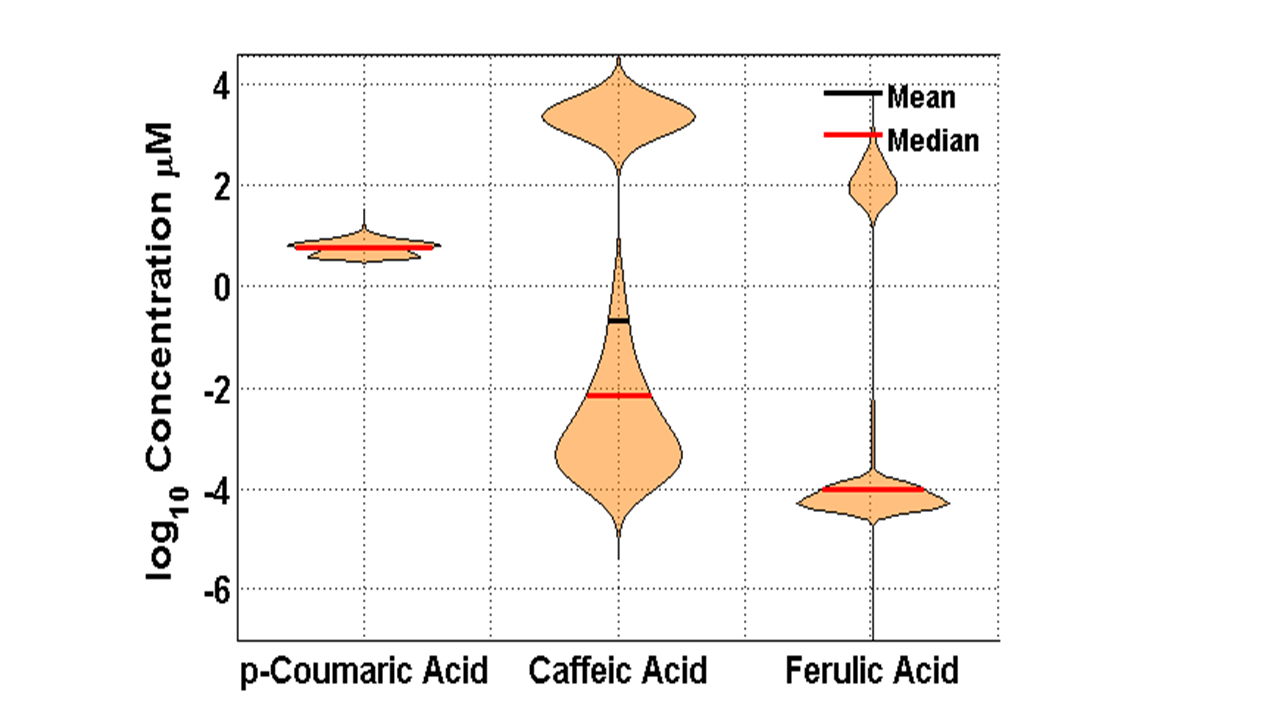

Supplement: S6 Fig — (TIF) [file pone.0193896.s006.tif]

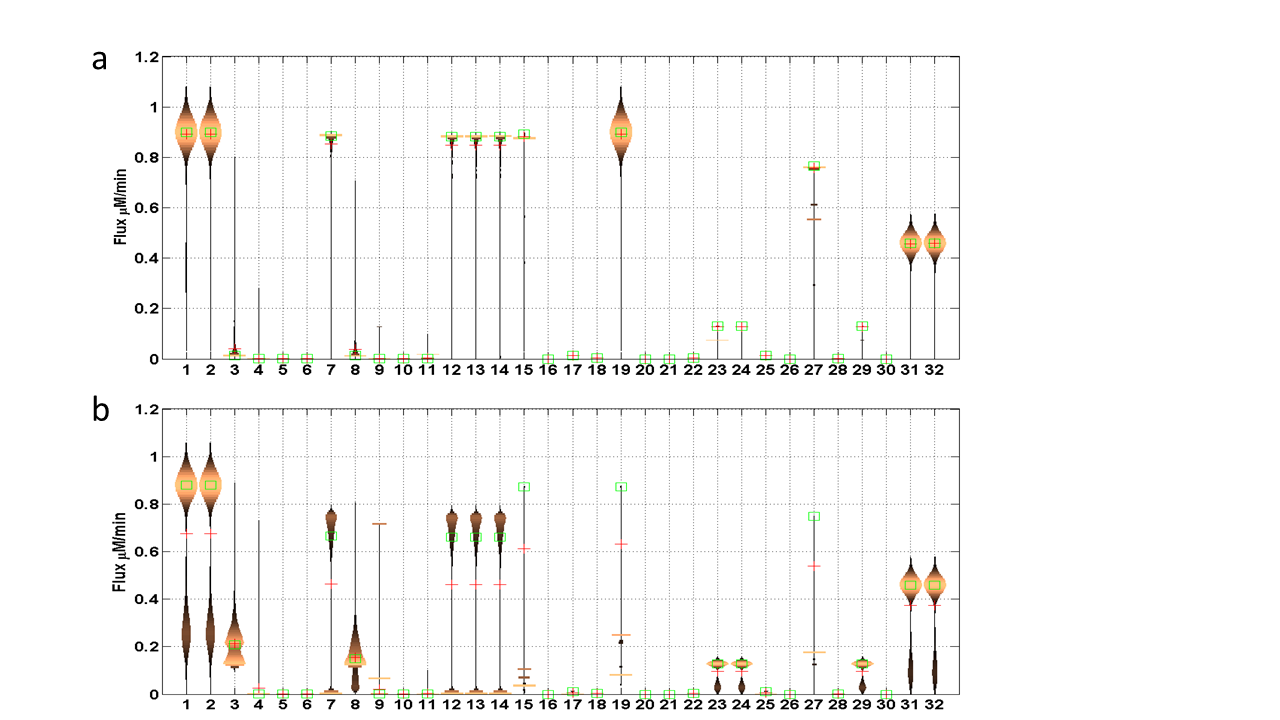

Supplement: S7 Fig — (a) The steady state flux distribution in the absence of Ptr4CL3-Ptr4CL5 complex. The green box represents the median steady state flux and the red ‘+’ sign represents the mean steady state flux values, (b) The steady state flux distribution in the presence of Ptr4CL3-Ptr4CL5 complex. The presence of complex induces a bimodal steady state flux distributions. The green box represents the median steady state flux and the red ‘+’ sign represents the mean steady state flux values. (TIF) [file pone.0193896.s007.tif]

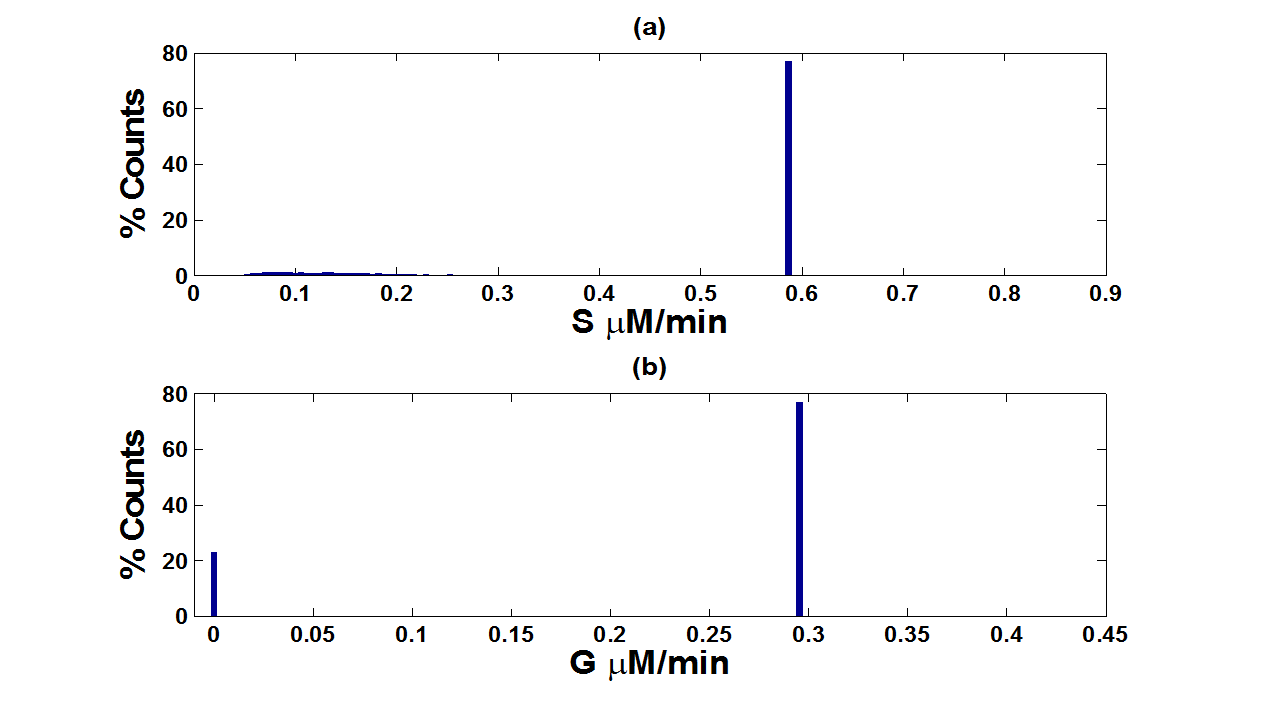

Supplement: S8 Fig — Variation of monolignol units resulting due to changes in levels of Ptr4CL3 and Ptr4CL5 concentrations in the presence of a complex: a) S and b) G. (TIF) [file pone.0193896.s008.tif]

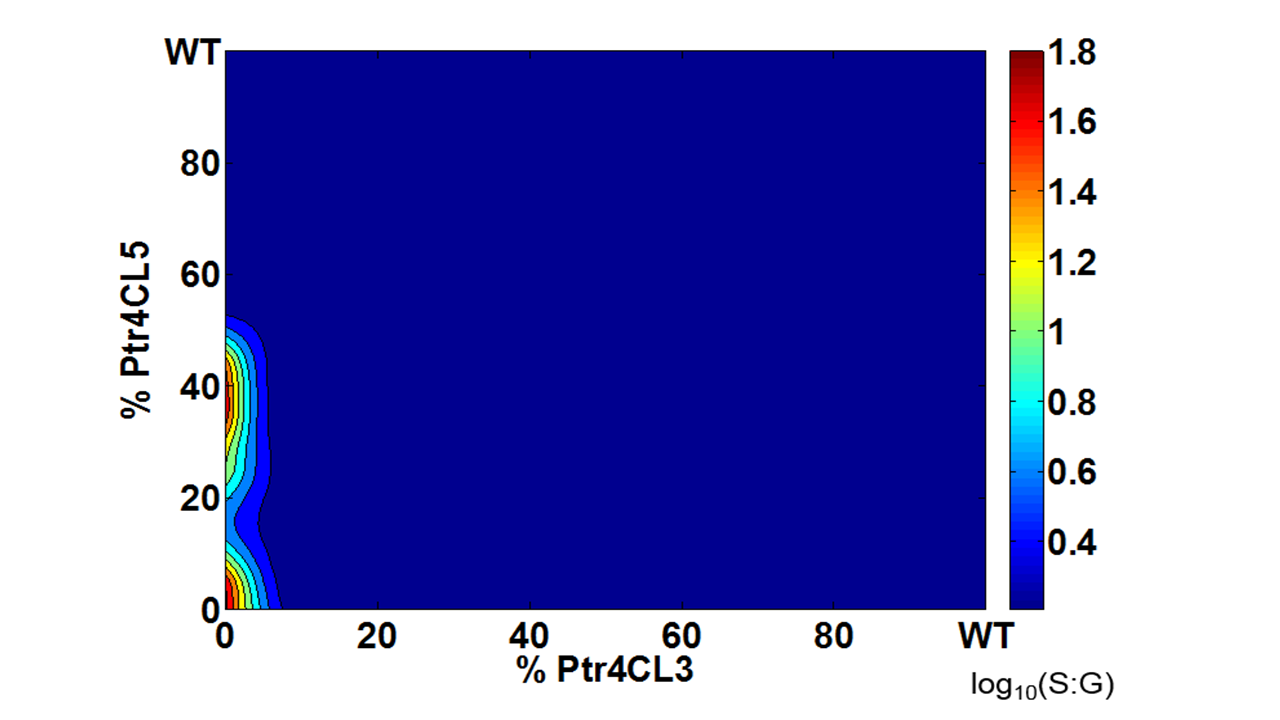

Supplement: S9 Fig — The color bar shows the variation of S/G ratio in log scale, where S/G ratio of 2 corresponds to a value of 0.3 in log scale. (TIF) [file pone.0193896.s009.tif]

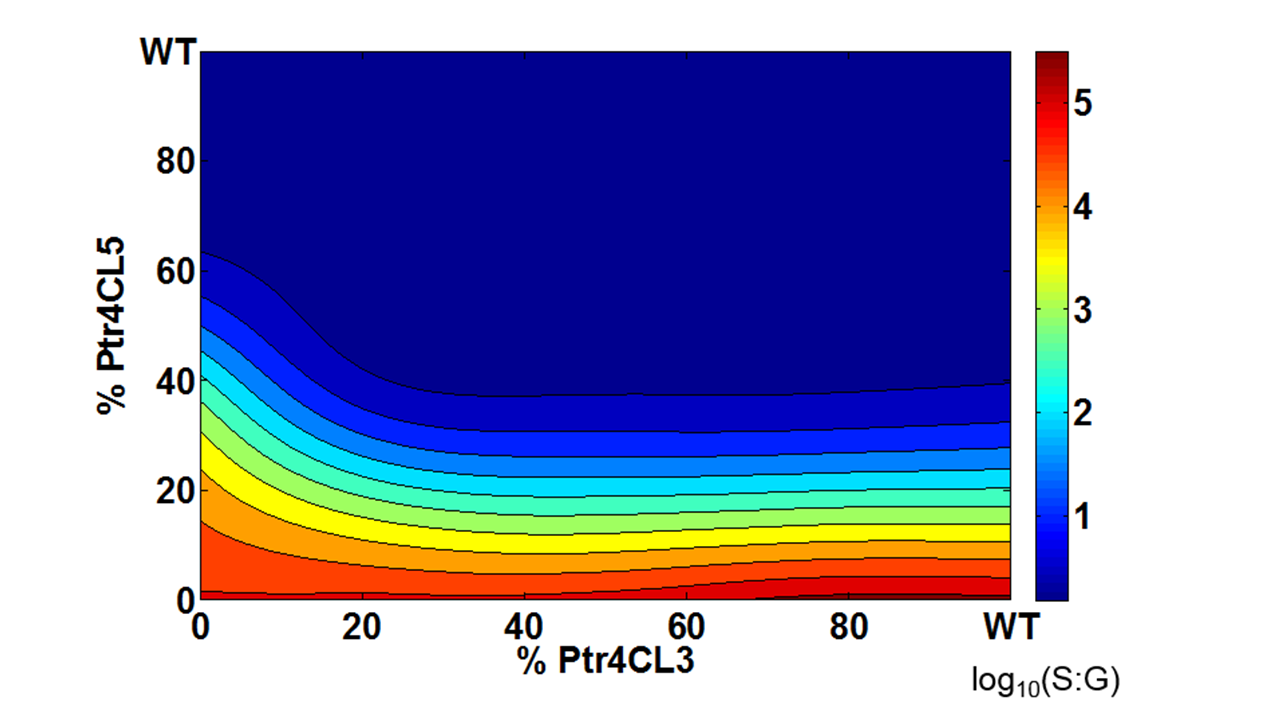

Supplement: S10 Fig — The color bar shows the variation of S/G ratio in log scale. (TIF) [file pone.0193896.s010.tif]
