# Supplementary material for: Assessing the impact of the 4CL enzyme complex on the robustness of monolignol biosynthesis using metabolic pathway analysis
Source: PLoS One. 2018 Mar 6;13(3):e0193896. doi: 10.1371/journal.pone.0193896 (PMC5839572; doi:10.1371/journal.pone.0193896)
Supplement: S1 Table — (DOCX) [file pone.0193896.s011.docx]

Supplemental Table 1: List of all the reactions involved in the monolignol biosynthesis pathway

| **Flux** | **Substrate** | **Product** | **Protein** |
| --- | --- | --- | --- |
| V_1_ | Phenylalanine | Cinnamic Acid | PAL |
| V_2_ | Cinnamic Acid | p-Coumaric acid | C4H |
| V_3_ | p-Coumaric acid | Caffeic Acid | C3H |
| V_4_ | Caffeic Acid | Ferulic Acid | COMT |
| V_5_ | Ferulic Acid | 5-Hydroxy ferulic Acid | CAld5H |
| V_6_ | 5-Hydroxy ferulic Acid | Sinapic Acid | COMT |
| V_7_ | p-coumaric acid | p-Coumary-CoA | 4CL |
| V_8_ | Caffeic Acid | Caffeoyl-CoA | 4CL |
| V_9_ | Ferulic Acid | Feruloyl-CoA | 4CL |
| V_10_ | 5-Hydroxy Ferulic Acid | 5-Hydroxy feruloyl-CoA | 4CL |
| V_11_ | Sinapic Acid | Sinapoyl-CoA | 4CL |
| V_12_ | p-Coumaryl-CoA | p-Coumaroyl Shikimic Acid | HCT |
| V_13_ | p-Coumaroyl Shikimic Acid | Caffeoyl Shikimic Acid | C3H |
| V_14_ | Caffeoyl Shikimic Acid | Caffeoyl-CoA | HCT |
| V_15_ | Caffeoyl-CoA | Feruloyl-CoA | CCoAOMT |
| V_16_ | 5-Hydroxy feruloyl-CoA | Sinapoyl-CoA | CCoAOMT |
| V_17_ | p-Coumaryl-CoA | p-Coumaraldehyde | CCR |
| V_18_ | Caffeoyl-CoA | Caffealdehyde | CCR |
| V_19_ | Feruloyl-CoA | Coniferaldehyde | CCR |
| V_20_ | 5-Hydroxy ceruloyl-CoA | 5-Hydroxy-coniferaldehyde | CCR |
| V_21_ | Sinapoyl-CoA | Sinapaldehyde | CCR |
| V_22_ | Caffealdehyde | Coniferaldehyde | COMT |
| V_23_ | Coniferaldehyde | 5-Hydroxy-coniferaldehyde | CAld5H |
| V_24_ | 5-Hydroxy-coniferaldehyde | Sinapaldehyde | COMT |
| V_25_ | p-Coumaraldehyde | p-Coumaryl Alcohol | CAD |
| V_26_ | Caffealdehyde | Caffeyl Alcohol | CAD |
| V_27_ | Coniferaldehyde | Coniferyl Alcohol | CAD |
| V_28_ | 5-Hydroxy-coniferaldehyde | 5-Hydroxy coniferyl Alcohol | CAD |
| V_29_ | Sinapaldehyde | Sinapyl Alcohol | CAD |
| V_30_ | Caffeyl Alcohol | Coniferyl Alcohol | COMT |
| V_31_ | Coniferyl Alcohol | 5-Hydroxy coniferyl Alcohol | CAld5H |
| V_32_ | 5-Hydroxy coniferyl Alcohol | Sinapyl Alcohol | COMT |

| **Variable** | **Substrate** |
| --- | --- |
| y1 | Phenylalanine |
| y2 | Cinnamic Acid |
| y3 | p-Coumaric acid |
| y4 | Caffeic Acid |
| y5 | Ferulic Acid |
| y6 | 5-Hydroxy ferulic Acid |
| y7 | Sinapic Acid |
| y8 | p-Coumaryl-CoA |
| y9 | p-Coumaroyl Shikimic Acid |
| y10 | Caffeoyl Shikimic Acid |
| y11 | Caffeoyl-CoA |
| y12 | Feruloyl-CoA |
| y13 | 5-Hydroxy feruloyl-CoA |
| y14 | Sinapoyl-CoA |
| y15 | p-Coumaraldehyde |
| y16 | Caffealdehyde |
| y17 | Coniferaldehyde |
| y18 | 5-Hydroxy-coniferaldehyde |
| y19 | Sinapaldehyde |
| y20 | p-coumaryl Alcohol |
| y21 | Caffeyl Alcohol |
| y22 | Coniferyl Alcohol |
| y23 | 5-Hydroxy coniferyl Alcohol |
| y24 | sinapyl Alcohol |
