# Supplementary material for: Assessing the impact of the 4CL enzyme complex on the robustness of monolignol biosynthesis using metabolic pathway analysis
Source: PLoS One. 2018 Mar 6;13(3):e0193896. doi: 10.1371/journal.pone.0193896 (PMC5839572; doi:10.1371/journal.pone.0193896)
Supplement: S2 Table — (PDF) [file pone.0193896.s012.pdf]

Metabolic Flux Model Equations

$$V1 = \frac{\mathit{kcat11}\,\mathrm{PtrPAL1}\,y_1}{y_1(1+\frac{y_4}{\mathit{kiu1}}) + \mathit{km11}(1+\frac{y_2}{\mathit{kic11}}+\frac{y_4}{\mathit{kic111}})} + \frac{\mathit{kcat12}\,\mathrm{PtrPAL2}\,y_1}{y_1(1+\frac{y_2}{\mathit{kiu12}}+\frac{y_4}{\mathit{kiu121}}) + \mathit{km12}(1+\frac{y_2}{\mathit{kic12}}+\frac{y_4}{\mathit{kic121}})} \quad (1)$$

$$+ \frac{\mathit{kcat13}\,\mathrm{PtrPAL3}\,y_1}{y_1(1+\frac{y_2}{\mathit{kiu13}}+\frac{y_4}{\mathit{kiu131}}) + \mathit{km13}(1+\frac{y_2}{\mathit{kic13}}+\frac{y_4}{\mathit{kic131}})} + \frac{\mathit{kcat14}\,\mathrm{PtrPAL45}\,y_1}{y_1(1+\frac{y_4}{\mathit{kiu14}}) + \mathit{km14}(1+\frac{y_2}{\mathit{kic14}}+\frac{y_4}{\mathit{kic141}})}$$

$$V2 = \frac{\mathit{kcat21}\,\mathrm{PtrC4H1}\,y_2}{y_2+\mathit{km21}} + \frac{\mathit{kcat22}\,\mathrm{PtrC4H2}\,y_2}{y_2+\mathit{km22}} \quad (2)$$

$$V3 = \frac{\mathit{kcat3}\,\mathrm{PtrC3H3}\,y_3}{y_3+\mathit{km3}} \quad (3)$$

$$V4 = \frac{\mathit{kcat4}\,\mathrm{PtrAldOMT2}\,y_4}{y_4(1+\frac{y_{16}}{\mathit{kiu41}}+\frac{y_{21}}{\mathit{kiu42}}+\frac{y_6}{\mathit{kiu43}}+\frac{y_{18}}{\mathit{kiu44}}+\frac{y_{23}}{\mathit{kiu45}}) + \mathit{km4}(1+\frac{y_{16}}{\mathit{kic41}}+\frac{y_{21}}{\mathit{kic42}}+\frac{y_6}{\mathit{kic43}}+\frac{y_{18}}{\mathit{kic44}}+\frac{y_{23}}{\mathit{kic45}})} \quad (4)$$

$$V5 = \frac{\mathit{kcat51}\,\mathrm{PtrCAld5H1}\,y_5}{y_5(1+\frac{y_{17}}{\mathit{kiu51}}) + \mathit{km51}(1+\frac{y_{17}}{\mathit{kic51}})} + \frac{\mathit{kcat52}\,\mathrm{PtrCAld5H2}\,y_5}{y_5(1+\frac{y_{17}}{\mathit{kiu52}}) + \mathit{km52}(1+\frac{y_{17}}{\mathit{kic52}})} \quad (5)$$

$$V6 = \frac{\mathit{kcat6}\,\mathrm{PtrAldOMT2}\,y_6}{y_6+\mathit{km6}} \quad (6)$$

$$V7 = \frac{\mathit{kcat71}\,\mathrm{Ptr4CL3}\,y_3}{\mathit{km71}(1+\frac{y_4}{\mathit{k73c1}}+\frac{y_5}{\mathit{k73c2}}+\frac{y_6}{\mathit{k73c3}}+\frac{y_7}{\mathit{k73c4}}+\frac{y_9}{\mathit{k73c5}}+\frac{y_{10}}{\mathit{k73c6}}) + y_3(1+\frac{y_9}{\mathit{k73u1}}+\frac{y_{10}}{\mathit{k73u2}})\cdots} \quad (7)$$

$$\cdots \overline{+\frac{3\mathit{km71}\,\mathrm{Ptr4CL3}^2\,\mathrm{Ptr4CL5}}{\mathit{K71}^3}(1+\frac{y_4}{\mathit{k75c1}}+\frac{y_5}{\mathit{k75c2}}+\frac{y_6}{\mathit{k75c3}}+\frac{y_7}{\mathit{k75c4}}+\frac{y_9}{\mathit{k75c5}}+\frac{y_{10}}{\mathit{k75c6}}+\frac{y_3}{\mathit{km72}}(1+\frac{y_4}{\mathit{k75u1}}+\frac{y_5}{\mathit{k75u2}}+\frac{y_6}{\mathit{k75u3}}+\frac{y_7}{\mathit{k75u4}}+\frac{y_9}{\mathit{k75u5}}+\frac{y_{10}}{\mathit{k75u6}})}$$

$$+ \frac{\mathit{kcat72}\,\mathrm{Ptr4CL5}\,y_3(1+\gamma\mathit{1}(\frac{\mathrm{Ptr4CL3}}{\mathit{K72}})^3)}{\mathit{km72}(1+\frac{y_4}{\mathit{k75c1}}+\frac{y_5}{\mathit{k75c2}}+\frac{y_6}{\mathit{k75c3}}+\frac{y_7}{\mathit{k75c4}}+\frac{y_9}{\mathit{k75c5}}+\frac{y_{10}}{\mathit{k75c6}}) + y_3(1+\frac{y_4}{\mathit{k75u1}}+\frac{y_5}{\mathit{k75u2}}+\frac{y_6}{\mathit{k75u3}}+\frac{y_7}{\mathit{k75u4}}+\frac{y_9}{\mathit{k75u5}}+\frac{y_{10}}{\mathit{k75u6}})(1+\frac{\mathrm{Ptr4CL3}}{\mathit{K72}})^3}$$

$$V8 = \frac{\mathit{kcat81}\,\mathrm{Ptr4Cl3}\,y_4}{\mathit{km81}(1+\frac{y_3}{\mathit{k83c1}}+\frac{y_5}{\mathit{k83c2}}+\frac{y_6}{\mathit{k83c3}}+\frac{y_9}{\mathit{k83c4}}) + y_4(1+\frac{y_9}{\mathit{k83u1}})\cdots} \quad (8)$$

$$\cdots \overline{+\frac{3\mathit{km81}\,\mathrm{Ptr4CL3}^2\,\mathrm{Ptr4CL5}}{\mathit{K81}^3}(1+\frac{y_3}{\mathit{k85c1}}+\frac{y_5}{\mathit{k85c2}}+\frac{y_6}{\mathit{k85c3}}+\frac{y_9}{\mathit{k85c4}}+\frac{y_4}{\mathit{km82}}(1+\frac{y_3}{\mathit{k85u1}}+\frac{y_5}{\mathit{k85u2}}+\frac{y_6}{\mathit{k85u3}}+\frac{y_7}{\mathit{k85u4}}+\frac{y_9}{\mathit{k85u5}}+\frac{y_4}{\mathit{k8is}}))}$$

$$+ \frac{\mathit{kcat82}\,\mathrm{Ptr4CL5}\,y_4(1+\gamma\mathit{2}(\frac{\mathrm{Ptr4CL3}}{\mathit{K82}})^3)}{\mathit{km82}(1+\frac{y_3}{\mathit{k85c1}}+\frac{y_5}{\mathit{k85c2}}+\frac{y_6}{\mathit{k85c3}}+\frac{y_9}{\mathit{k85c4}}) + y_4(1+\frac{y_3}{\mathit{k85u1}}+\frac{y_5}{\mathit{k85u2}}+\frac{y_6}{\mathit{k85u3}}+\frac{y_7}{\mathit{k85u4}}+\frac{y_9}{\mathit{k85u5}}+\frac{y_4}{\mathit{k8is}})(1+(\frac{\mathrm{Ptr4CL3}}{\mathit{K82}})^3)}$$

$$V9 = \frac{\mathit{kcat91}\,\mathrm{Ptr4CL3}\,y_5}{\mathit{km91}(1 + \frac{y_3}{\mathit{k93c1}} + \frac{y_4}{\mathit{k93c2}} + \frac{y_6}{\mathit{k93c3}} + \frac{y_7}{\mathit{k93c4}}) + y_5} + \frac{\mathit{kcat92}\,\mathrm{Ptr4CL5}\,y_5}{\mathit{km92}(1 + \frac{y_3}{\mathit{k95c1}} + \frac{y_4}{\mathit{k95c2}} + \frac{y_6}{\mathit{k95c3}} + \frac{y_7}{\mathit{k95c4}}) + y_5(1 + \frac{y_4}{\mathit{k95u1}} + \frac{y_6}{\mathit{k95u2}} + \frac{y_7}{\mathit{k95u3}})} \quad (9)$$

$$V10 = \frac{\mathit{kcat101}\,\mathrm{Ptr4CL3}\,y_6}{\mathit{km101} + y_6} + \frac{\mathit{kcat102}\,\mathrm{Ptr4CL5}\,y_6}{\mathit{km102} + y_6} \quad (10)$$

$$V11 = \frac{\mathit{kcat111}\,\mathrm{Ptr4CL5}\,y_7}{\mathit{km111} + y_7} \quad (11)$$

$$V12 = \frac{\mathit{kcat121}\,\mathrm{PtrHCT1}\,y_8}{y_8 + \mathit{km121}} + \frac{\mathit{kcat122}\,\mathrm{PtrHCT6}\,y_8}{y_8 + \mathit{km122}} \quad (12)$$

$$V12R = \frac{\mathit{kcat121R}\,\mathrm{PtrHCT1}\,y_9}{y_9 + \mathit{km121R}} + \frac{\mathit{kcat122R}\,\mathrm{PtrHCT6}\,y_9}{y_9 + \mathit{km122R}} \quad (13)$$

$$V13 = \frac{\mathit{kcat131}\,\mathrm{PtrC3H3}\,y_9}{y_9 + \mathit{km131}} \quad (14)$$

$$V14 = \frac{\mathit{kcat141}\,\mathrm{PtrHCT1}\,y_{10}}{y_{10} + \mathit{km141}} + \frac{\mathit{kcat142}\,\mathrm{PtrHCT6}\,y_{10}}{y_{10} + \mathit{km142}} \quad (15)$$

$$V14R = \frac{\mathit{kcat141R}\,\mathrm{PtrHCT1}\,y_{11}}{y_{11} + \mathit{km141R}} + \frac{\mathit{kcat142R}\,\mathrm{PtrHCT6}\,y_{11}}{y_{11} + \mathit{km142R}} \quad (16)$$

$$V15 = \frac{\mathit{kcat151}\,\mathrm{PtrCCoAOMT1}\,y_{11}}{y_{11} + \mathit{km151}} + \frac{\mathit{kcat152}\,\mathrm{PtrCCoAOMT2}\,y_{11}}{y_{11} + \mathit{km152}} + \frac{\mathit{kcat153}\,\mathrm{PtrCCoAOMT3}\,y_{11}}{y_{11} + \mathit{km153}} \quad (17)$$

$$V16 = 0 \quad (18)$$

$$V17 = \frac{\mathit{kcat181}\,\mathrm{PtrCCR2}\,y_8}{y_8 + \mathit{km181}} \quad (19)$$

$$V18 = \frac{\mathit{kcat191}\,\mathrm{PtrCCR2}\,y_{11}}{y_{11} + \mathit{km191}} \quad (20)$$

$$V19 = \frac{\mathit{kcat201}\,\mathrm{PtrCCR2}\,y_{12}}{y_{12} + \mathit{km201}} \quad (21)$$

$$V20 = 0 \quad (22)$$

$$V21 = 0 \quad (23)$$

$$V22 = \frac{kcat241 \mathrm{PtrAldOMT2} y_{16}}{y_{16}(1 + \frac{y_{18}}{kiu241} + \frac{y_{16}}{kis241}) + km241(1 + \frac{y_4}{kic241} + \frac{y_6}{kic242} + \frac{y_{18}}{kic243} + \frac{y_{23}}{kic244})} \tag{24}$$

$$V23 = \frac{kcat251 \mathrm{PtrCAld5H1} y_{17}}{y_{17} + km251} + \frac{kcat252 \mathrm{PtrCAld5H2} y_{17}}{y_{17} + km252} \tag{25}$$

$$V24 = \frac{kcat261 \mathrm{PtrAldOMT2} y_{18}}{y_{18}(1 + \frac{y_{21}}{kiu261} + \frac{y_{18}}{kis261}) + km261(1 + \frac{y_{16}}{kic261} + \frac{y_{21}}{kic262} + \frac{y_{23}}{kic263})} \tag{26}$$

$$V25 = \frac{kcat271 \mathrm{PtrCAD1} y_{15}}{y_{15} + km271} \tag{27}$$

$$V26 = 0 \tag{28}$$

$$V27 = \frac{kcat291 \mathrm{PtrCAD1} y_{17}}{y_{17}(1 + \frac{y_{19}}{kiu291}) + km291(1 + \frac{y_{19}}{kic291})} + \frac{kcat292 \mathrm{PtrCAD2} y_{17}}{y_{17} + km292} \tag{29}$$

$$V28 = \frac{kcat281 \mathrm{PtrCAD1} y_{18}}{y_{18} + km281} \tag{30}$$

$$V29 = \frac{kcat311 \mathrm{PtrCAD1} y_{19}}{y_{19}(1 + \frac{y_{17}}{kiu311}) + km311(1 + \frac{y_{17}}{kic311})} + \frac{kcat312 \mathrm{PtrCAD2} y_{19}}{y_{19} + km312} \tag{31}$$

$$V30 = \frac{kcat331 \mathrm{PtrAldOMT2} y_{21}}{y_{21}(1 + \frac{y_{16}}{kiu331} + \frac{y_{18}}{kiu332} + \frac{y_{23}}{kiu333} + \frac{y_{21}}{kis331}) + km331(1 + \frac{y_{16}}{kic331} + \frac{y_{18}}{kic332})} \tag{32}$$

$$V31 = \frac{kcat341 \mathrm{PtrCAld5H1} y_{22}}{y_{22}(1 + \frac{y_{17}}{kiu341}) + km341(1 + \frac{y_{17}}{kic341})} + \frac{kcat342 \mathrm{PtrCAld5H2} y_{22}}{y_{22} + km342(1 + \frac{y_{17}}{kic342})} \tag{33}$$

$$V32 = \frac{kcat351 \mathrm{PtrAldOMT2} y_{23}}{y_{23}(1 + \frac{y_{23}}{kis351}) + km351} \tag{34}$$

$$V33 = V25 \tag{35}$$

$$V34 = V27 + V30 - V31 \tag{36}$$

$$V35 = V29 + V32 \tag{37}$$

$$\frac{\mathrm{d}y_1}{\mathrm{d}t} = V0 - V2 \tag{38}$$

$$\frac{\mathrm{d}y_2}{\mathrm{d}t} = V1 - V2 \tag{39}$$

$$\frac{\mathrm{d}y_3}{\mathrm{d}t} = V2 - V3 - V7 \tag{40}$$

$$\frac{\mathrm{d}y_4}{\mathrm{d}t} = V3 - V4 - V8 \tag{41}$$

$$\frac{\mathrm{d}y_5}{\mathrm{d}t} = V4 - V5 - V9 \tag{42}$$

$$\frac{\mathrm{d}y_6}{\mathrm{d}t} = V5 - V6 - V10 \tag{43}$$

$$\frac{\mathrm{d}y_7}{\mathrm{d}t} = V6 - V11 \tag{44}$$

$$\frac{\mathrm{d}y_8}{\mathrm{d}t} = V7 - V12 \tag{45}$$

$$\frac{\mathrm{d}y_9}{\mathrm{d}t} = V12 - V13 \tag{46}$$

$$\frac{\mathrm{d}y_{10}}{\mathrm{d}t} = V13 - V14 \tag{47}$$

$$\frac{\mathrm{d}y_{11}}{\mathrm{d}t} = V8 + V14 - V15 - V18 \tag{48}$$

$$\frac{\mathrm{d}y_{12}}{\mathrm{d}t} = V9 + V15 - V19 \tag{49}$$

$$\frac{\mathrm{d}y_{13}}{\mathrm{d}t} = V10 - V16 - V20 \tag{50}$$

$$\frac{\mathrm{d}y_{14}}{\mathrm{d}t} = V11 + V16 - V21 \tag{51}$$

$$\frac{\mathrm{d}y_{15}}{\mathrm{d}t} = V12 - V25 \tag{52}$$

$$\frac{\mathrm{d}y_{16}}{\mathrm{d}t} = V18 - V22 - V26 \tag{53}$$

$$\frac{\mathrm{d}y_{17}}{\mathrm{d}t} = V19 + V22 - V23 - V27 \tag{54}$$

$$\frac{\mathrm{d}y_{18}}{\mathrm{d}t} = V20 + V23 - V24 - V28 \tag{55}$$

$$\frac{\mathrm{d}y_{19}}{\mathrm{d}t} = V21 + V24 - V29 \tag{56}$$

$$\frac{\mathrm{d}y_{20}}{\mathrm{d}t} = V25 - V33 \tag{57}$$

$$\frac{\mathrm{d}y_{21}}{\mathrm{d}t} = V26 - V30 \tag{58}$$

$$\frac{\mathrm{d}y_{22}}{\mathrm{d}t} = V27 + V30 - V31 - VG \tag{59}$$

$$\frac{\mathrm{d}y_{23}}{\mathrm{d}t} = V28 + V31 - V32 \tag{60}$$

$$\frac{\mathrm{d}y_{24}}{\mathrm{d}t} = V29 + V32 - VS \tag{61}$$
