# Supplementary material for: Assessing the impact of the 4CL enzyme complex on the robustness of monolignol biosynthesis using metabolic pathway analysis
Source: PLoS One. 2018 Mar 6;13(3):e0193896. doi: 10.1371/journal.pone.0193896 (PMC5839572; doi:10.1371/journal.pone.0193896)
Supplement: S3 Table — (PDF) [file pone.0193896.s013.pdf]

Metabolic Flux Model Equations with Complex

$$
\begin{aligned}
V7 = {} & \frac{\mathit{kcat71}\,\mathrm{Ptr4CL3}\,y_3}{\mathit{km71}(1+\frac{y_4}{\mathit{k73c1}}+\frac{y_5}{\mathit{k73c2}}+\frac{y_6}{\mathit{k73c3}}+\frac{y_7}{\mathit{k73c4}}+\frac{y_9}{\mathit{k73c5}}+\frac{y_{10}}{\mathit{k73c6}})+y_3(1+\frac{y_9}{\mathit{k73u1}}+\frac{y_{10}}{\mathit{k73u2}})}\cdots \\
& \cdots\frac{}{+\frac{3\mathit{km71}\,\mathrm{Ptr4CL3}^2\mathrm{Ptr4CL5}}{\mathit{K71}^3}(1+\frac{y_4}{\mathit{k75c1}}+\frac{y_5}{\mathit{k75c2}}+\frac{y_6}{\mathit{k75c3}}+\frac{y_7}{\mathit{k75c4}}+\frac{y_9}{\mathit{k75c5}}+\frac{y_{10}}{\mathit{k75c6}}+\frac{y_3}{\mathit{km72}}(1+\frac{y_4}{\mathit{k75u1}}+\frac{y_5}{\mathit{k75u2}}+\frac{y_6}{\mathit{k75u3}}+\frac{y_7}{\mathit{k75u4}}+\frac{y_9}{\mathit{k75u5}}+\frac{y_{10}}{\mathit{k75u6}})} \\
& +\frac{\mathit{kcat72}\,\mathrm{Ptr4CL5}\,y_3(1+\gamma\mathit{1}(\frac{\mathrm{Ptr4CL3}}{\mathit{K72}})^3)}{\mathit{km72}(1+\frac{y_4}{\mathit{k75c1}}+\frac{y_5}{\mathit{k75c2}}+\frac{y_6}{\mathit{k75c3}}+\frac{y_7}{\mathit{k75c4}}+\frac{y_9}{\mathit{k75c5}}+\frac{y_{10}}{\mathit{k75c6}})+y_3(1+\frac{y_4}{\mathit{k75u1}}+\frac{y_5}{\mathit{k75u2}}+\frac{y_6}{\mathit{k75u3}}+\frac{y_7}{\mathit{k75u4}}+\frac{y_9}{\mathit{k75u5}}+\frac{y_{10}}{\mathit{k75u6}})(1+\frac{\mathrm{Ptr4CL3}}{\mathit{K72}})^3}
\end{aligned} \tag{1}
$$

$$
\begin{aligned}
V8 = {} & \frac{\mathit{kcat81}\,\mathrm{Ptr4Cl3}\,y_4}{\mathit{km81}(1+\frac{y_3}{\mathit{k83c1}}+\frac{y_5}{\mathit{k83c2}}+\frac{y_6}{\mathit{k83c3}}+\frac{y_9}{\mathit{k83c4}})+y_4(1+\frac{y_9}{\mathit{k83u1}})}\cdots \\
& \cdots\frac{}{+\frac{3\mathit{km81}\,\mathrm{Ptr4CL3}^2\mathrm{Ptr4CL5}}{\mathit{K81}^3}(1+\frac{y_3}{\mathit{k85c1}}+\frac{y_5}{\mathit{k85c2}}+\frac{y_6}{\mathit{k85c3}}+\frac{y_9}{\mathit{k85c4}}+\frac{y_4}{\mathit{km82}}(1+\frac{y_3}{\mathit{k85u1}}+\frac{y_5}{\mathit{k85u2}}+\frac{y_6}{\mathit{k85u3}}+\frac{y_7}{\mathit{k85u4}}+\frac{y_9}{\mathit{k85u5}}+\frac{y_4}{\mathit{k8is}}))} \\
& +\frac{\mathit{kcat82}\,\mathrm{Ptr4CL5}\,y_4(1+\gamma\mathit{2}(\frac{\mathrm{Ptr4CL3}}{\mathit{K82}})^3)}{\mathit{km82}(1+\frac{y_3}{\mathit{k85c1}}+\frac{y_5}{\mathit{k85c2}}+\frac{y_6}{\mathit{k85c3}}+\frac{y_9}{\mathit{k85c4}})+y_4(1+\frac{y_3}{\mathit{k85u1}}+\frac{y_5}{\mathit{k85u2}}+\frac{y_6}{\mathit{k85u3}}+\frac{y_7}{\mathit{k85u4}}+\frac{y_9}{\mathit{k85u5}}+\frac{y_4}{\mathit{k8is}})(1+(\frac{\mathrm{Ptr4CL3}}{\mathit{K82}})^3)}
\end{aligned} \tag{2}
$$

$$
\begin{aligned}
V9 = {} & \frac{\mathit{kcat91}\,\mathrm{Ptr4CL3}\,y_5}{\mathit{km91}(1+\frac{y_3}{\mathit{k93c1}}+\frac{y_4}{\mathit{k93c2}}+\frac{y_6}{\mathit{k93c3}}+\frac{y_7}{\mathit{k93c4}})+y_5} \\
& +\frac{\mathit{kcat92}\,\mathrm{Ptr4CL5}\,y_5}{\mathit{km92}(1+\frac{y_3}{\mathit{k95c1}}+\frac{y_4}{\mathit{k95c2}}+\frac{y_6}{\mathit{k95c3}}+\frac{y_7}{\mathit{k95c4}})+y_5(1+\frac{y_4}{\mathit{k95u1}}+\frac{y_6}{\mathit{k95u2}}+\frac{y_7}{\mathit{k95u3}})}
\end{aligned} \tag{3}
$$

$$
V10 = \frac{\mathit{kcat101}\,\mathrm{Ptr4CL3}\,y_6}{\mathit{km101}+y_6}+\frac{\mathit{kcat102}\,\mathrm{Ptr4CL5}\,y_6}{\mathit{km102}+y_6} \tag{4}
$$

$$
V11 = \frac{\mathit{kcat111}\,\mathrm{Ptr4CL5}\,y_7}{\mathit{km111}+y_7} \tag{5}
$$

Metabolic Flux Model Equations without Complex

$$V7 = \frac{\mathit{kcat71}\,\mathrm{Ptr4CL3}\,y_3}{\mathit{km71}(1 + \frac{y_4}{\mathit{k73c1}} + \frac{y_5}{\mathit{k73c2}} + \frac{y_6}{\mathit{k73c3}} + \frac{y_7}{\mathit{k73c4}} + \frac{y_9}{\mathit{k73c5}} + \frac{y_{10}}{\mathit{k73c6}}) + y_3(1 + \frac{y_9}{\mathit{k73u1}} + \frac{y_{10}}{\mathit{k73u2}})} \tag{6}$$

$$+ \frac{\mathit{kcat72}\,\mathrm{Ptr4CL5}\,y_3}{\mathit{km72}(1 + \frac{y_4}{\mathit{k75c1}} + \frac{y_5}{\mathit{k75c2}} + \frac{y_6}{\mathit{k75c3}} + \frac{y_7}{\mathit{k75c4}} + \frac{y_9}{\mathit{k75c5}} + \frac{y_{10}}{\mathit{k75c6}}) + y_3(1 + \frac{y_4}{\mathit{k75u1}} + \frac{y_5}{\mathit{k75u2}} + \frac{y_6}{\mathit{k75u3}} + \frac{y_7}{\mathit{k75u4}} + \frac{y_9}{\mathit{k75u5}} + \frac{y_{10}}{\mathit{k75u6}})}$$

$$V8 = \frac{\mathit{kcat81}\,\mathrm{Ptr4Cl3}\,y_4}{\mathit{km81}(1 + \frac{y_3}{\mathit{k83c1}} + \frac{y_5}{\mathit{k83c2}} + \frac{y_6}{\mathit{k83c3}} + \frac{y_9}{\mathit{k83c4}}) + y_4(1 + \frac{y_9}{\mathit{k83u1}})} \tag{7}$$

$$+ \frac{\mathit{kcat82}\,\mathrm{Ptr4CL5}\,y_4}{\mathit{km82}(1 + \frac{y_3}{\mathit{k85c1}} + \frac{y_5}{\mathit{k85c2}} + \frac{y_6}{\mathit{k85c3}} + \frac{y_9}{\mathit{k85c4}}) + y_4(1 + \frac{y_3}{\mathit{k85u1}} + \frac{y_5}{\mathit{k85u2}} + \frac{y_6}{\mathit{k85u3}} + \frac{y_7}{\mathit{k85u4}} + \frac{y_9}{\mathit{k85u5}} + \frac{y_4}{\mathit{k8is}})}$$

$$V9 = \frac{\mathit{kcat91}\,\mathrm{Ptr4CL3}\,y_5}{\mathit{km91}(1 + \frac{y_3}{\mathit{k93c1}} + \frac{y_4}{\mathit{k93c2}} + \frac{y_6}{\mathit{k93c3}} + \frac{y_7}{\mathit{k93c4}}) + y_5} \tag{8}$$

$$+ \frac{\mathit{kcat92}\,\mathrm{Ptr4CL5}\,y_5}{\mathit{km92}(1 + \frac{y_3}{\mathit{k95c1}} + \frac{y_4}{\mathit{k95c2}} + \frac{y_6}{\mathit{k95c3}} + \frac{y_7}{\mathit{k95c4}}) + y_5(1 + \frac{y_4}{\mathit{k95u1}} + \frac{y_6}{\mathit{k95u2}} + \frac{y_7}{\mathit{k95u3}})}$$

$$V10 = \frac{\mathit{kcat101}\,\mathrm{Ptr4CL3}\,y_6}{\mathit{km101} + y_6} + \frac{\mathit{kcat102}\,\mathrm{Ptr4CL5}\,y_6}{\mathit{km102} + y_6} \tag{9}$$

$$V11 = \frac{\mathit{kcat111}\,\mathrm{Ptr4CL5}\,y_7}{\mathit{km111} + y_7} \tag{10}$$
